# Heritability of overlapping impulsivity and compulsivity dimensional phenotypes

**DOI:** 10.1038/s41598-020-71013-x

**Published:** 2020-09-01

**Authors:** Jeggan Tiego, Samuel R. Chamberlain, Ben J. Harrison, Andrew Dawson, Lucy Albertella, George J. Youssef, Leonardo F. Fontenelle, Murat Yücel

**Affiliations:** 1 Brain, Mind and Society Research Hub, Monash Biomedical Imaging, Turner Institute for Brain and Mental Health, and School of Psychological Sciences, Monash University, 770 Blackburn Road, Clayton, VIC 3800 Australia; 2 Department of Psychiatry, University of Cambridge, Cambridge, UK; 3 Cambridge Peterborough NHS Foundation Trust, Cambridge, UK; 4 Department of Psychiatry, Melbourne Neuropsychiatry Centre, University of Melbourne and Melbourne Health, Melbourne, Australia; 5 School of Psychology, Faculty of Health, Centre for Social and Early Emotional Development, Deakin University, Geelong, Australia; 6 Centre for Adolescent Health, Murdoch Children’s Research Institute, Melbourne, Australia; 7 Obsessive, Compulsive, and Anxiety Spectrum Research Program, Institute of Psychiatry, Federal University of Rio de Janeiro, Rio de Janeiro, Brazil; 8 D’Or Institute for Research and Education, Rio de Janeiro, Brazil

**Keywords:** Psychology, Human behaviour

## Abstract

Impulsivity and compulsivity are traits relevant to a range of mental health problems and have traditionally been conceptualised as distinct constructs. Here, we reconceptualised impulsivity and compulsivity as partially overlapping phenotypes using a bifactor modelling approach and estimated heritability for their shared and unique phenotypic variance within a classical twin design. Adult twin pairs (N = 173) completed self-report questionnaires measuring psychological processes related to impulsivity and compulsivity. We fitted variance components models to three uncorrelated phenotypic dimensions: a general impulsive–compulsive dimension; and two narrower phenotypes related to impulsivity and obsessiveness.There was evidence of moderate heritability for impulsivity (A^2^ = 0.33), modest additive genetic or common environmental effects for obsessiveness (A^2^ = 0.25; C^2^ = 0.23), and moderate effects of common environment (C^2^ = 0.36) for the general dimension, This general impulsive–compulsive phenotype may reflect a quantitative liability to related mental health disorders that indexes exposure to potentially modifiable environmental risk factors.

## Introduction

Psychiatric research is transitioning away from traditional categorisations of mental disorders as discrete diagnostic entities towards empirically based, dimensional models of psychopathology^1,2^. An aim of dimensional psychiatry is to identify aetiological mechanisms that transcend traditional diagnostic categories^3^. Dimensional approaches leverage quantitative methods to capture covariation in psychopathology symptoms and relevant psychological processes into a smaller number of continuously distributed phenotypic dimensions in the population^4^. Mental disorders may reflect the extreme end of the spectrum of quantitative traits that are expressed throughout the population and extend into subclinical and non-clinical ranges^5,6^. By capturing phenotypic variation across the full spectrum of symptom severity, dimensional models are better positioned to identify shared aetiological mechanisms and the common genetic architecture of psychiatric disorders^2,6,7^.

Hierarchical organisation is an important feature of dimensional models, such that distributed traits relevant to psychopathology can be understood at varying level of generality and specificity^1,8^. Psychiatric phenotypes can be organised into hierarchical dimensions using bifactor models within a structural equation modelling framework, which enable common variance across phenotypic traits to be separated from unique within-trait variance^4^. Partitioning phenotypic variance into common and specific dimensions facilitates novel insights into the operation of aetiological mechanisms, such as identification of broad versus narrow genetic risk factors^8,9^.

Impulsivity and compulsivity are psychiatric phenotypes implicated in the aetiology and maintenance of impulse control, obsessive–compulsive, and addictive and related disorders^10,11^. Impulsivity is a multifaceted construct that describes a predisposition to rapid unplanned actions without preplanning or forethought and without consideration of potential adverse consequences^12,13^. Compulsivity is likewise a multifaceted construct that describes patterns of behaviour that are repeated despite undesirable consequences^11,14^. Impulsivity and compulsivity have historically been conceptualised as opposite ends of a single underlying spectrum, extending from risk-seeking (i.e. reward-seeking) to risk-avoidance (i.e. harm-avoidance)^15,16^. More recent conceptualisations suggest considerable phenotypic and neurobiological overlap between impulsivity and compulsivity^10,17,18^. This overlap may reflect dysfunction in top-down, goal-directed cognitive control mechanisms^19–24^ and be particularly relevant to the aetiology and maintenance of impulse control, obsessive–compulsive, and addictive and related disorders^25,26^.

The overlapping dimensional phenotypes model operationalises impulsivity and compulsivity as empirically-related continuous dimensions using a bifactor model within a structural equation modelling (SEM) framework^22^. We fitted a bifactor model to data obtained from self-report questionnaires measuring psychological processes related to impulsivity, obsessive beliefs, and intolerance of uncertainty in an unselected community sample of 587 adults. The model included three uncorrelated and normally distributed dimensions: (1) a general factor, reflecting a combination of high general impulsivity, intolerance of uncertainty, and obsessive beliefs; (2) an ‘Impulsivity’ factor, capturing residual variance specific to sensation seeking, high emotion-based rash action (i.e. impulsive behaviours in response to positive and negative emotions), and a lack of premeditation; and (3) a ‘Compulsivity’ factor, reflecting residual variance specific to intolerance of uncertainty, obsessive beliefs, perfectionism, perceived responsibility, and sensitivity to threat. We found that the general dimension explained the most variance in co-occurring impulse control problems, including alcohol misuse, problem gambling, and compulsive shopping, as well as obsessive–compulsive-related problems, including problematic use of the internet, binge eating, and obsessive–compulsive symptoms. The Impulsivity and Compulsivity factors explained additional unique variance in impulse control problems and obsessive–compulsive-related problems, respectively.

We have since replicated the overlapping dimensional phenotypes model in an independent longitudinal study sample using a different measure of impulsivity and compulsivity and showed that the three, empirically distinct phenotypic dimensions were uniquely associated with a number of risk factors and antecedents^27^. This model has also been extended to incorporate participants from an imaging study with diagnosed obsessive–compulsive disorder and gambling disorder, as well as healthy controls. Resting state functional Magnetic Resonance Imaging data were analysed using dynamic causal modeling^28^. Higher levels of the general dimension, combining psychological processes related to impulsivity and compulsivity, were associated transdiagnostically with lower bottom-up effective connectivity in the dorsal circuit and greater bottom-up effective connectivity in the ventral circuit^21^. These findings are consistent with the theory that impulsivity and compulsivity reflect dysfunctional changes in cortical-striatal-thalamic-cortical circuits leading to impairments in top-down cognitive control underlying a range of psychiatric disorders^18–20,29,30^. This empirical overlap between psychological processes related to impulsivity and compulsivity is thus a potentially informative model for understanding impulse control, obsessive–compulsive, and addiction-related problems, in terms of shared aetiology and transdiagnostic treatment approaches.

Obtaining estimates of heritability, the proportion of variance in a phenotype attributable to genetic versus environmental effects, has implications for understanding the aetiological mechanisms linking dimensional phenotypes to related psychological disorders^31^. The results of initial quantitative genetics studies may also inform subsequent genome-wide association studies and interventions research^32,33^. The classical twin study design represents a suitable methodology for testing hypotheses of heritability, particularly for complex traits^32,34^. Embedded within an SEM framework, the classical twin design allows researchers to estimate the degree of population variability  in a phenotype that is attributable to the influence of genetic (additive [A^2^] or dominance [D^2^] effects), common environment [C^2^], and unique environment [E^2^] effects by comparing within-trait correlations between monozygotic and dizygotic twins^35,36^. It also allows for the fit of competing models to be directly compared using statistical criteria^35,36^. Heritability (h^2^) represents the proportion of phenotypic variance attributable to genetic effects, divided by the total variance explained by genetic, common and unique environment, as well as unexplained error variance (h^2^ = A^2^ + D^2^/A^2^ + D^2^ + C^2^ + E^2^)^35,37^.

Previous studies have sought to quantify the genetic contribution to impulsivity using behavioural and self-report measures within a classic twin study methodology. Broad-sense heritability estimates of between 0.38 and 0.50 have been reported for impulsivity with no contribution of common environment across the lifespan (i.e. ADE or AE models)^38–40^. Compulsivity has not been researched as thoroughly, nor is it as well-understood, as impulsivity^11,14,18^. Until recently, researchers have tended to conceptualise compulsivity with reference to obsessive compulsive disorder (OCD), which has been considered the ‘archetypal’ compulsive disorder^16,41^. Thus, several studies have examined the heritability of OCD symptom dimensions and symptoms of related obsessive–compulsive spectrum disorders^42–46^. These studies have revealed modest to strong heritability for obsessive–compulsive symptoms, generally with no effect of common environment (i.e. AE models)^47–49^. The broad consensus from these studies is that impulsivity and compulsivity are best attributed to modest to strong genetic effects, with the rest attributable to unique environment and measurement error.

The purpose of the current study was to investigate the heritability of both shared and unique phenotypic variance in impulsivity and compulsivity. Understanding the genetic and environmental influences on the overlap between impulsivity and compulsivity, as well as their independent phenotypic variance, may provide useful insights into the aetiology of impulse control, obsessive–compulsive, and addiction-related disorders, as well as facilitate future large-scale genetic studies. Based on previous studies examining the impulsivity phenotype and OCD-related symptoms in adult twin samples, we hypothesised that additive genetic effects models with no common environment effects (i.e. AE models) would provide the best fit to covariance data obtained from monozygotic and dizygotic twins. We expected that heritability estimates obtained from these AE models for impulsivity and compulsivity phenotypes would be modest to moderate (h^2^ ~ 0.30–0.50). Given the paucity of previous research directly examining the phenotypic overlap of psychological processes related to impulsivity and compulsivity, we tentatively hypothesised an AE model and modest to moderate heritability for the general factor (i.e. AE model).

## Results

Twins from each pair were randomly assigned (i.e. twin 1 or twin 2) to separate subsamples for preliminary analyses and cross-validation of the results prior to being combined for the variance components models and heritability analyses. Descriptive statistics for the Urgency, Premeditation (lack of), Perseverance (lack of), Sensation Seeking, and Positive Urgency (UPPS-P) Impulsivity Behavior Scale, Intolerance of Uncertainty Scale 12-item version (IUS-12), and Obsessive Beliefs Questionnaire 44-item version (OBQ-44) subscales are provided in Tables S1 and S2 Supplementary Information. Results of first-order confirmatory factor analysis (CFA) of the UPPS-P, IUS-12, and OBQ-44 subscale structure in the twin 1 subsample, along with cross-validation in the twin 2 subsample with invariance testing, are described in Supplementary Information and presented in Supplementary Tables S3–S6 online. Information functions obtained from the first-order CFA of the UPPS-P, IUS-12, and OBQ-44 based on competing factor models (i.e. published subscale structure versus bifactor models) are displayed in Fig. S1–S10 Supplementary Information, and reveal the precision of measurement across the latent trait continuum for each factor solution based on item response theory. Model fit statistics displayed in Tables S3–S6, in combination with the information functions in Fig. S1–S10, showed that four-, two- and three-factor models that reproduced the published subscale structures of the UPPS-P, IUS-12, and OBQ-44 provided a better fit to the data compared to competing models. Subscale raw scores were thus used as indicators (i.e. dependent/observed variables) for testing competing second-order models of impulsivity and compulsivity.

### Bifactor model of impulsivity and compulsivity

Subscale raw scores were entered as the indicator variables for the bifactor model. Negative Urgency and Positive Urgency items were combined into a single ‘Urgency’ subscale based on a four-factor model solution of the UPPS-P (see Supplementary Table S3 online). A bifactor model was specified after Tiego et al. (2018) with all nine variables loading on a general factor, the four UPPS-P subscales loading on an ‘Impulsivity’ group factor, and the IUS-12 and OBQ-44 subscales loading on a ‘Compulsivity’ group factor. The three factors were specified as orthogonal dimensions (i.e. factor intercorrelations were constrained to zero). The Lack of Perseverance (UPPS-P) subscale failed to load on the general factor; the Sensation Seeking (UPPS-P) subscale did not load on the Impulsivity factor, and the Desire for Predictability and an Active Engagement in Seeking Certainty and Paralysis of Cognition and Action in the Face of Uncertainty (IUS-12) subscales did not load on the Compulsivity factor. These loadings were not freely estimated in the next iteration of the model. Several error covariances were freely estimated to obtain model fit; all corrected for multiple post hoc comparisons (i.e. Benjamini–Hochberg false discovery rate [q = 0.05])^50^.

**Figure 1:**
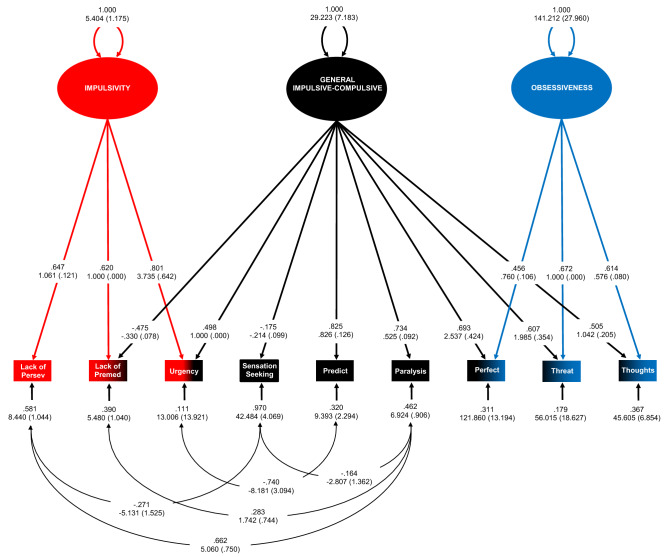
Bifactor model of psychological processes related to impulsivity and compulsivity in the twin 1 subsample. Model fit statistics were χ^2^(17) = 30.359, p = 0.082; RMSEA = 0.057 [90%CI = 0.021, .089]; CFI = 0.983; SRMR = 0.064. All five error covariances were adjusted for multiple post hoc comparisons (B-H p = 0.039). N = 242. Model figures are displayed using symbols from the McArdle-McDonald reticular action model^131^. Observed (also measured or manifest) variables are represented as rectangles. Latent variables are represented as ellipses: (1) factors (or constructs) are represented as large ellipses; (2) error variances for observed variables are symbolised with small ellipses. Double-headed, curved arrows pointing to factors are the latent variable variances. Straight, single-headed arrows from large ellipses to observed variables reflect factor loadings. Straight, single-headed arrows pointing from small ellipses to measured variables are the (i.e. error) variances in the variables not explained by the factor. Curved, double-headed arrows between small ellipses are error covariances. Factor scaling was performed using the reference variable method, with the unstandardised loading estimate of one measured variable for each factor set to one. Fully standardised parameter estimates appear above with unstandardised parameter estimates and bootstrapped standard errors (10,000 posterior draws) in brackets below. *Lack of Persev* lack of perseverance raw subscale scores (UPPS-P), *Lack of Premed* lack of premeditation raw subscale scores (UPPS-P), *Urgency* summed raw scores on the positive urgency and negative urgency subscales (UPPS-P), *Sensation Seeking* sensation seeking raw subscale scores (UPPS-P), *Predict* desire for predictability and an active engagement in seeking certainty subscale raw scores (IUS-12), *Paralysis* paralysis of cognition and action in the face of uncertainty subscale raw scores (IUS-12), *Perfect* perfectionism and intolerance of uncertainty subscale raw scores (OBQ-44), *Threat* responsibility and threat estimation subscale raw scores (OBQ-44), *Thoughts* importance and control of thoughts subscale raw scores (OBQ-44). Figure created in Microsoft Excel 2016, Office desktop (16.0.12624.20424) 64-bit.

The final model is displayed in Fig. 1 (χ^2^(17) = 30.359, p = 0.082; RMSEA = 0.057 [90%CI = 0.021, 0.089]; CFI = 0.983; SRMR = 0.064). The pattern and strengths of factor loadings were interpretable as representing three distinct phenotypes: (1) a general ‘Impulsive–Compulsive’ –dimension with higher levels reflecting greater emotion-based rash action (i.e. urgency), intolerance of uncertainty, and obsessive beliefs; (2) Impulsivity—higher levels of this phenotype capturing more emotion-based rash action (i.e. urgency) in combination with lower levels of premeditation and perseverance; and (3) Obsessiveness—higher levels of this phenotype reflecting greater presence of obsessive beliefs related to responsibility, threat estimation, perfectionism and intolerance of uncertainty, and the importance and control of thoughts. In contrast to the original model, this second group factor had only loadings from the three OBQ-44 subscales representing different types of obsessive beliefs, and no loadings indicative of increased premeditation and perseverance^22^. We therefore named this factor ‘Obsessiveness’ to reflect a narrower empirical construct and interpretation than the previous ‘Compulsivity’ factor.

Model fit statistics for the competing one-, two-, and three-factor models are provided in Table S7 Supplementary Material. The two- and three-factor models failed to provide a good fit to the data. Two of nine subscales, Sensations Seeking and Lack of Perseverance, had to be dropped to facilitate fit for the unidimensional model, thus compromising construct representation and criterion-related validity of the factors^51^. The hypothesised bifactor model therefore provided the best fit to the data. The model was cross-validated in the twin 2 subsample using invariance testing. The intercorrelation matrix for the subscale scores in twin 1 and twin 2 subsamples is displayed in Table S8. The results of invariance testing are provided in Table 1 and Table S9. Full configural, weak, strong, and partial strict invariance (i.e. only two indicators were not measured with the same level of precision) were demonstrated in the twin 2 subsample along with equivalence of factor variances (Table 1) and means (Table S9). These results indicated that the model had very stable measurement properties and the three phenotypes were being measured equivalently and could be compared meaningfully across twin pairs.

**Table 1:** Results of invariance testing for the bifactor model of impulsivity and compulsivity phenotypes in the twin 1 and twin 2 subsamples.

Model	df	χ^2^	p	RMSEA (90% CI)	CFI	SRMR	Δdf	Δχ^2^	Δp
Configural invariance	35	76.310	0.266	0.070 (0.048–0.091)	0.973	0.060			
Weak invariance	46	87.844	0.345	0.061 (0.041–0.080)	0.973	0.066	11	11.534	0.400
Strong invariance^a^	55	98.454	0.340	0.057 (0.038–0.075)	0.972	0.065	9	10.610	0.303
Partial strict invariance^b^	61	106.176	0.372	0.055 (0.037–0.072)	0.971	0.082	6	7.722	0.259
Equality of factor variances	64	107.317	0.390	0.053 (0.035–0.070)	0.972	0.086	3	1.141	0.767

N = 486 (Twin 1 subsample n = 241; Twin 2 subsample n = 245).

*df* degress of freedom, *χ*^*2*^ Chi square value for test of model fit using full information maximum likelihood estimation, *p* significance value of the chi square test statistic, *RMSEA* root mean square error of approximation, *CI* confidence interval, *SRMR* standardised root mean residual, *CFI* comparative fit index, *Δdf* delta degrees of freedom, *Δχ*^*2*^ delta chi square, *Δp* significance value of the delta chi square test statistic.

^a^Equality of latent means were tested after strong invariance was determined (impulsive–compulsive [z = 0.441, p = 0.660]; impulsivity [z = − 0.319, p = 0.750]; compulsivity [z = − 1.333, p = 0.183]).

^b^Partial strict invariance—the error variances for the Urgency (θ_δ_ = 56.185, SE = 10.284, p < 0.001), Importance and Control of Thoughts (θ_δ_ = 157.541, SE = 88.171, p = 0.074), and the Paralysis of Cognition and Action (θ_δ_ = 16.600, SE = 2.095, p < 0.001) subscales were freely estimated in the twin 2 subsample to obtain acceptable fit. Error covariances also differed between subsamples and were therefore unconstrained and freely estimated within groups.

### Heritability analyses

Within-trait and between trait subscale correlations for monozygotic and dizygotic twins are provided in Table S10 and S11, respectively. The twin 1 and twin 2 subsamples were combined and the bifactor model specified to generate factor score estimates using the regression method^52–54^. Factor score determinacies were high for the general Impulsive–Compulsive, Impulsivity, and Obsessiveness m phenotypes, respectively (ρ = 0.914, 0.861, 0.814), indicating that the factor score estimates provided relatively accurate measurement of individual differences on the three latent variables^52,53^. Additionally, the factor score estimates were only weakly correlated (general factor with Impulsivity r = 0.001, p = 0.981 and Compulsivity r = 0.239, p < 0.001; Impulsivity with Compulsivity r = 0.175, p < 0.001), suggesting that the orthogonality of the phenotypes in the bifactor model had largely been preserved (i.e. correlational preserving) and that the factor score estimates were not being unduly contaminated by variance from the other phenotypes (i.e. univocality)^52,53^.

**Figure 2:**
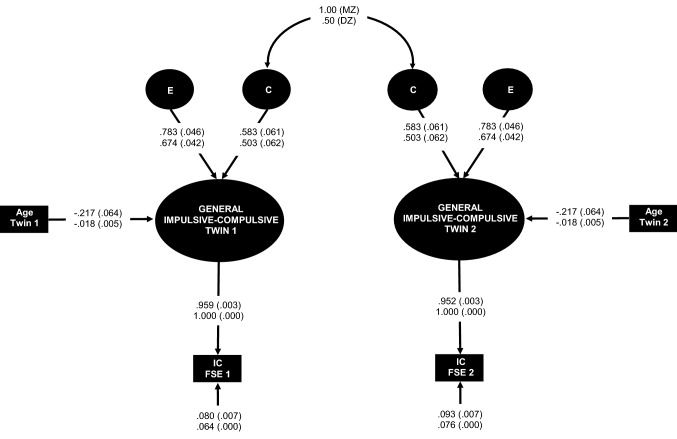
Common environment and unique environment plus error (CE) [χ^2^(13) = 17.226, p = 0.189; BIC = 901.560. H_i_ (Pr | D) = 0.727] variance components model for the Impulsive–Compulsive phenotype. *MZ* monozygotic twins, *DZ* dizygotic twins, *E* unique environment plus measurement error variance component, *C* common environment variance component, *Age* age in years, *IC FSE 1* twin 1 subsample Impulsive–Compulsive factor score estimates, *IC FSE 2* twin 2 subsample Impulsive–Compulsive factor score estimates. Fully standardised parameter estimates with standard errors in brackets appear above. Unstandardised parameter estimates with standard errors in brackets appear below. Figure created in Microsoft Excel 2016, Office desktop (16.0.12624.20424) 64-bit.

**Figure 3:**
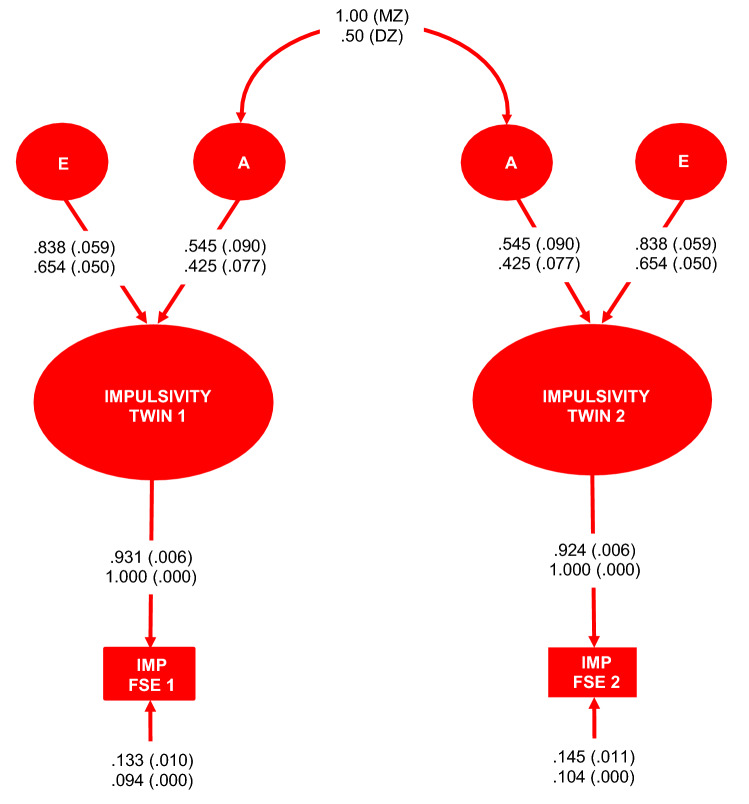
Additive genetic and unique environment plus error (AE) [χ^2^(6) = 5.177, p = 0.521; BIC = 873.819; H_i_ (Pr | D) =  0.604]; variance components model for the Impulsivity phenotype. *MZ* monozygotic twins, *DZ* dizygotic twins, *E* unique environment plus measurement error variance component, *A* additive genetic variance component, *Age* age in years, *IMP FSE 1* twin 1 subsample impulsivity factor score estimates, *IMP FSE 2* twin 2 subsample impulsivity factor score estimates. Fully standardised parameter estimates with standard errors in brackets appear above. Unstandardised parameter estimates with standard errors in brackets appear below. Figure created in Microsoft Excel 2016, Office desktop (16.0.12624.20424) 64-bit.

**Figure 4:**
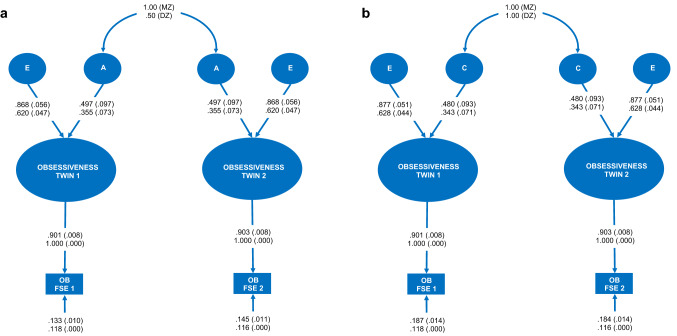
(**a**) Additive genetic and unique environment plus error (AE) [χ^2^(6) = 7.583, p = 0.270; BIC = 835.777; H_i_ (Pr | D) =  0.274]; and (**b**) common environment and unique environment plus error (CE) [χ^2^(6) = 7.426, p = 0.283; BIC = 835.621; H_i_ (Pr | D) =  0.296] variance components models for the obsessiveness phenotype. *MZ* monozygotic twins, *DZ* dizygotic twins, *E* unique environment variance component, *A* additive genetic variance component, *C* common environment variance component, *Age* age in years, *OB FSE 1* twin 1 subsample obsessiveness factor score estimates, *OB FSE 2* twin 2 subsample obsessiveness factor score estimates. Fully standardised parameter estimates with standard errors in brackets appear above. Unstandardised parameter estimates with standard errors in brackets appear below. Figure created in Microsoft Excel 2016, Office desktop (16.0.12624.20424) 64-bit.

The distributions of the factor scores estimates were examined separately for the twin 1 and twin 2 subsamples (see Fig. S11–S13 Supplementary Information online) and screened for univariate outliers. Two outliers were removed for the general dimension from the twin 2 subsample and recoded as missing data^55^. The factor scores estimates for the twin 1 and twin 2 subsamples were entered as single-indicator latent variables with error variance fixed to reflect the inverse of the product of the factor determinacy and the variance of the subsample [θ_δ_ = (1 – ρ)*σ^2^]^52,56^. This approach enabled greater control of error in the estimation process of individual differences in the latent dimensional phenotypes using the factor score estimates. Sex and age were initially included as regressors in the models but did not predict variance in Impulsivity or Compulsivity. Age, but not sex, was a meaningful predictor of twin differences in the general Impulsive–Compulsive dimension and was therefore included in all the variance components models for this phenotype. Nested variance components models were generated for all three phenotypic dimensions and directly compared for statistical fit to the data using Bayesian statistics. The results are summarised in Table 2. The pattern of covariances between monozygotic and dizygotic twin pairs was best represented by a CE model for the general Impulsive–Compulsive dimension (see Fig. 2), an AE model for Impulsivity (see Fig. 3), and an AE or CE model for Obsessiveness (see Fig. 4A,B). Alternative models are displayed for Obsessiveness because there was insufficient evidence for adjudicating between them based on Bayesian statistics^57,58^. The proportion of population variability in the general Impulsive–Compulsive phenotype attributabed to common environment was 36%. Heritability of the Impulsivity phenotype was estimated as 0.33 for the AE model. Heritability of the Obsessiveness phenotype was estimated as 0.25 in the AE model. Conversely, common environment was estimated to explain 23% of the population variability in Obsessiveness in the CE model.

**Table 2:** Fit statistics for competing variance components models for the impulsive–compulsive, impulsivity, and obsessiveness phenotypes.

Phenotype	Model	− 2*LL	df	χ^2^	P	BIC	H_i_ (Pr | D)
Impulsive–compulsive^a^	ACE	− 437.897	12	28.101	0.141	906.714	0.055
	AE	− 439.103	13	19.639	0.105	903.973	0.217
	CE^d^	− 437.897	13	17.226	0.189	901.560	0.727
	E	− 447.563	14	36.558	0.001	915.739	0.001
Impulsivity	ACE	− 426.603	5	5.177	0.395	878.972	0.046
	AE^d^	− 426.603	6	5.177	0.521	873.819	0.604
	ADE	− 424.014	5	3.699	0.594	877.494	0.096
	CE	− 428.187	6	8.346	0.214	876.987	0.124
	E	− 430.712	7	13.396	0.063	876.884	0.130
Obsessiveness	ACE	− 407.493	5	7.404	0.192	840.752	0.030
	AE^b^	− 407.582	6	7.583	0.270	835.777	0.356
	CE^c^	− 407.504	6	7.426	0.283	835.621	0.385
	E	− 410.602	7	13.622	0.058	836.663	0.229

− 2*LL − 2 × log likelihood, *df* degrees of freedom for the chi square test statistic, *χ*^*2*^ chi square test of model fit, *p* probability of the chi square test statistic, *BIC* Bayesian information criterion, *H*_*i*_*(Pr|D)* Bayesian conditional posterior probability of model H_i_ compared with H_k_ models, *A* additive genetic effects, *C* common environment effects, *E* unique environment effects + error variance, *D* dominance genetic effects.

^a^Age included in all models as a regressor for the impulsive-compulsive  phenotype.

^b^Bayesian posterior probability of AE compared to CE = [H_0_ (Pr | D) = 0.481].

^c^Bayesian posterior probability of CE compared to AE = [H_1_ (Pr | D) = 0.519].

^d^Preferred model based on weight of evidence.

## Discussion

Impulsivity and compulsivity are candidate psychiatric phenotypes with potential transdiagnostic utility in dimensional psychiatry, particularly in relation to impulse control and obsessive–compulsive and related disorders, as well as substance and behavioural addictions^10,24,59^. Previous twin modeling studies have explored the heritability of broadly defined traits of impulsivity and compulsivity in isolation and obtained modest to strong heritability estimates^38,42–45^. The present study extended the focus of existing literature by examining impulsivity and compulsivity in concert, specifically their phenotypic overlap^21–23^. We operationalised these latent phenotypes as self-reported psychological processes related to different forms of impulsivity, intolerance of uncertainty, and obsessive beliefs. Using the classical twin design, we found that a CE variance components model provided the best fit to the pattern of covariances between monozygotic and dizygotic twins for the general dimension, reflecting a combination of intolerance of uncertainty, obsessive beliefs, and a tendency towards emotion-based rash action. The results suggested a modest effect of common environment on individual differences in this phenotype (C^2^ = 0.36). This finding is notable in that several previous behavioural genetic studies have found negligible effects of common environment on broadly defined traits of impulsivity and compulsivity^38,42–46^. Thus, this phenotypic dimension may provide a window into the mechanisms contributing to the co-occurrence of impulsive and compulsive behavioural tendencies, which appear to have a distinct aetiology, as indicated by the finding of a moderate sized effect of the common environment. Importantly, these results do not conclusively demonstrate negligible genetic effects, because what is decomposed into common environment in the variance components model can partly reflect genetic effects, or genotype-environment correlations^33,36,60^.

The general Impulsive–Compulsive factor here defined may be compared with the ‘Behavioral Disinhibition’ latent phenotype described in previous behavioural and molecular genetics literature^61–65^. Behavioural Disinhibition describes a spectrum of psychological disorders, temperament/personality traits, and related behaviours representing a common inability to inhibit impulsive actions^62,64^. Among the facets reflecting Behavioral Disinhibition are diagnoses of conduct disorder, attention deficit hyperactivity disorder, antisocial personality disorder, as well as temperament traits of novelty seeking, and low constraint^61–63^. Behavioral Disinhibition has been found to be moderately to highly heritable, with zero to modest effects of the common environment^62,65,66^. This latent phenotype has become the focus of behavioural and molecular genetic studies because it was constructed to capture and quantify a common liability to impulsive action that is highly heritable and confers a vulnerability to the development of a range of impulsive and compulsive disorders^67,68^.

Here, we have sought to quantify the influence of genetic and environmental effects on the common and unique variance of self-reported psychological processes related to impulsivity, intolerance of uncertainty, and obsessive beliefs because of their putative role in the development of impulsive and compulsive psychopathology. Our model explicitly incorporates psychological processes related to risk-avoidance (e.g. threat estimation; paralysis of cognition and action in the face of uncertainty) that have traditionally been viewed as anti-correlated with the risk-taking and reward-seeking central to the construct of impulsivity^15,16^. Only more recently has the potential phenotypic and neurobiological overlap of impulsivity and compulsivity been explored and explicitly tested^18,59^. Furthermore, the bifactor modelling approach we have used here enables both the shared *and* unique variance in psychological processes related to impulsivity and compulsivity to be examined, as opposed to only the shared variance captured by a common factor model, such as that typically used to model Behavioral Disinhibition^70^. We have previously shown that these unique phenotypic dimensions related to impulsivity and compulsivity are associated with distinct antecedents and neurobiological substrates, as well as explaining additional variance in impulse-control and obsessive–compulsive related problems^27,71,22^. However, by virtue of capturing shared variance across addiction-related problems and dimensions of personality and temperament such as constraint, the Behavioral Disinhibition construct likely incorporates variance related to compulsivity. Thus, research that directly and empirically compares these constructs within the same sample would be needed to establish the discriminant validity of the general Impulsive–Compulsive dimension defined in this study from the Behavioral Disinhibition construct previously established in the behavioural genetics literature^10,22^.

Interestingly, there were weak negative associations with age, suggesting that levels of the general phenotype tended to be slightly lower in older compared with younger participants. The general factor in the model explained comparatively more variance in the IUS-12 and OBQ-44 subscales relative to the UPPS-P subscales. This indicates that the general phenotype largely reflects intolerance of uncertainty and obsessive beliefs, in addition to emotion-based rash action, and lack of premeditation. Uncertainty intolerance has been shown to decrease with age, partly due to changes in the perceived adaptive and functional benefits of worry^72^. Older individuals may be more competent and motivated towards selecting situations and environments that foster stability, predictability, and well-being^73,74^. Importantly, intolerance of uncertainty and obsessive beliefs are amenable to therapeutic change, suggesting potential intervention strategies for reducing what could be a general liability for impulsive and compulsive problems^75–77^.

This study extends previous behavioural genetic research by examining heritability of the unique variance in impulsivity and obsessiveness following removal of the empirical overlap between these phenotypes. We found that the proportion of variance in the impulsivity phenotype attributable to additive genetic effects (A^2^ = 0.33) was comparable to that reported in a meta-analysis of behavioural genetic studies when examining questionnaire data (A^2^ = 0.29—0.37), but less than broad sense heritability estimates (i.e. A^2^ + D^2^ = 0.41—0.69) obtained from the best-fitting ADE models^38^. In our study, the ADE model did not provide improvement in fit compared to the AE model, indicating that the combination of additive and dominance genetic effects was unnecessary to account for the stronger covariances in monozygotic compared to dizygotic twins. This may suggest that the impulsivity phenotype in our model reflects a narrower trait associated with smaller genetic effects than the broadly defined trait of impulsivity generally investigated in the literature. However, impulsivity is a multifaceted construct and there is yet no consensus on the number and operationalisation of what may be several phenotypically and genetically distinct traits^12,13,38^. In particular, there is poor convergence between self-report and behavioural measure of impulsivity, suggesting these methods assess at least partly dissociable constructs^78^. Further studies would be required to clarify how impulsivity, as we have defined and measured it, relates to the different variations of the impulsivity construct treated in the literature.

The heritability estimate obtained from the AE model for the Obsessiveness factor was modest (A^2^ = 0.25) and slightly lower than estimates reported in twin modeling studies of obsessive–compulsive and related disorders (A^2^ ~ 0.30)^42–45^. These results again may reflect differences in the operationalisation and measurement of these compulsivity-related constructs. As previously noted, compulsivity has not been as well-defined nor thoroughly investigated as impulsivity^11,14,18^. Researchers have tended to operationalise compulsivity with reference to OCD and related obsessive–compulsive spectrum disorders^16,41^. This divergence in conceptualisation and measurement may explain the difference in heritability estimates. Additionally, previous studies examining the heritability of obsessive–compulsive and related disorders have not controlled for phenotypic overlap with impulsivity. An intimate relationship has been proposed between predisposing levels of impulsivity and the subsequent development of compulsivity, suggesting these traits may have common genetic and environmental aetiological mechanisms^19^. Parsing distinct compulsivity-related variance from overlapping variance with impulsivity may explain the discrepant results. Furthermore, evidence for the AE model was equivocal and a CE model provided a comparable fit to the pattern of covariances between monozygotic and dizygotic twins. This may suggest that there is no contribution of genetic effects following removal of the phenotypic overlap with impulsivity. Follow-up studies with more statistical power are required to adjudicate between the AE and CE models and address this question.

Heritability has been posited as an important criterion for evaluating candidate psychiatric endophenotypes^79,80^. Endophenotypes are a key concept in dimensional psychiatry, referring to latent (not visible to unaided observation) quantitative traits that index liability for psychiatric disorders^31,81^. Identification of psychiatric endophenotypes is intended to increase conceptual clarity and precision of measurement in dimensional psychiatry^80,82^. However, psychiatric endophenotypes that index environmental risk may be just as useful for understanding the aetiology of mental disorders^31^. As a so-called “environmental endophenotype” (Kendler & Neale, 2010, p. 795), the general Impulsive–Compulsive dimension in the current study, capturing intolerance of uncertainty, obsessiveness, and predisposition towards emotion-based rash action, could provide a quantitative index of liability to various disorders that may be attributable to one or more identifiable environmental mechanisms. Importantly, these environmental influences may be modifiable with implications for intervention and treatment of impulse control, obsessive–compulsive, and addictive disorders.

It is also possible that this general dimension partly reflects genotype-environment correlations, suggesting some contribution of genetics to individual differences in the overlap of psychological processes related to impulsivity and compulsivity^33,60^. Nevertheless, the modest contributions of shared environment to this phenotype is an important validation of the general factor in our model. The general factor in bifactor models has been criticised as a common method or response bias factor, or other forms of random error variance^83,84^. The present results suggest that the general factor in the overlapping dimensional phenotypes model reflects meaningful phenotypic variance, rather than merely random error variance, as would be indicated by a better fit for an E compared to a CE variance components model.The current study also demonstrates replicability of this model in an independent sample, and extends on previous studies by using a more parsimonious measurement approach (see Supplementary Material for further details)^21,22^.

A criticism of bifactor models is their tendency to provide superior fit compared to competing models because they flexibly accommodate patterns of covariance regardless of population data structure^70,85,86^. However, results of model testing support use of the bifactor model as a theoretically consistent and empirically plausible representation of the current data. The pattern and strength of factor loadings were consistent with the presence of a robust general factor and two residual group factors, all with statistically significant variance estimates^70,87^. There were no illogical or near boundary parameter estimates and the standard errors were all small and within a sensible range^88,89^. Only a few theoretically-plausible freely estimated error covariances were required to obtain close fit to the data, all statistically significant when adjusting for multiple post hoc comparisons using the false discovery rate^50^. Thus, there were no signs in the model parameter estimates, or global and local fit indices, that the bifactor model was misspecified. When added to our previous findings of meaningful links between these three phenotypes with antecedent risks, symptoms of psychopathology, and neurobiology, we feel confident that the bifactor model is a plausible and useful representation of individual difference variance in self-reported psychological processes related to impulsivity and compulsivity^22,27,71^.

We found that a CE variance components model provided a superior fit to the pattern of covariances between monozygotic and dizygotic twins than an AE model for the general dimension. However, evidence for the CE over the AE model using Bayesian statistics was relatively weak by conventional standards^57,58,90^. Similarly, there was insufficient statistical evidence to adjudicate between the AE and CE models for the Obsessiveness phenotype. Further studies with larger numbers of twin pairs are needed to replicate the findings and adequately compare the competing models^91,92^. In particular, statistical power to detect additive genetic effects is reduced as the ratio of dizygotic to monozygotic twin pairs decreases^91,92^. This reduces confidence in the finding that the CE model provided a better fit to the data than the AE model for the general phenotype. Our results should therefore be considered tentative and with reference to these methodological limitations. Future studies of the heritability of this general phenotype, reflecting intolerance of uncertainty, obsessiveness, and emotion-based rash action, will require much larger samples and a higher ratio of dizygotic to monozygotic twin pairs to ensure adequate power to adjudicate between AE and CE models. With larger sample sizes, multivariate twin models would be an important extension of this study and could investigate whether the genetic and environmental effects common to comorbid disorders is partly attributable to variance in these three phenotypes, suggesting a common transdiagnostic aetiology^45,93^.

The strength of genetic effects on the expression of complex traits, such as impulsivity, changes across the lifespan and notably has been shown to increase with age, possibly due to gene-environment interactions^38,94^. In our sample, there was a difference of medium sized effect in the mean age of monozygotic [*M* = 38.60, *SD* = 10.42] and dizygotic [*M* = 44.60, *SD* = 9.35] twins used in the variance components models [*t* (171) = 3.64, *p* < 0.001; Cohen’s d = 0.61; ΔM = − 6.00 (95% CI − 2.75, − 9.25)]. Model parameter estimates may have been affected if the relationship between age and the phenotypes differs markedly across the age range. There were minimal differences in the sex composition for monozygotic and dizygotic twin pairs in the final sample used to for the variance components models [χ(1) = 1.651, p = 0.199, n = 344]. Furthermore, there has been mixed evidence for sex effects in the genetic and environmental aetiology of phenotypes related to normal personality and psychopathology, including impulsivity^38,40,49,94–96^. Nevertheless, much larger samples would be required to test sex limitation models^91^.

We operationalised impulsivity using the UPPS-P Impulsive Behaviour Scale, a popular multi-dimensional model used in the wider personality and psychopathology literature to measure impulsivity^98–101^. Nevertheless, not all researchers agree on the validity or utility of differentiating between five facets of impulsivity and broad consensus has converged on far fewer distinct impulsivity constructs relevant to psychopathology^102–104^. We recapitulated our original finding that Negative Urgency and Positive Urgency were indistinguishable at the level of latent variables and could be collapsed into a unitary ‘Urgency’ construct, which also parallels the second-order ‘Emotion-Based Rash Action’ factor previously reported for the short-version of the UPPS-P^22,105^. Negative Urgency and Positive Urgency have often been subsumed under the common ‘Urgency’ construct in relation to addiction research^106,107^. Interestingly, Cyders et al. (2014) also reported a second-order ‘Deficits in Conscientiousness’ factor consisting of loadings from the Lack of Premeditation and Lack of Perseverance factors. Sensation Seeking was found to be empirically distinct from these otherwise conceptually similar impulsivity constructs, similar to the findings reported here and elsewhere previously^108^. Further work is needed to understand how the three target phenotypes in our model relate to the different conceptualisations of impulsivity and compulsivity in the literature^18^.

The compulsivity-related phenotype measured in the present study likely reflects an ‘Obsessiveness’ dimension rather than a broader compulsivity construct. A previous study found that a unidimensional ‘Obsessionality’ factor explained the majority of variance in OCD symptom domains and was partly heritable, obtaining a similar estimate of additive genetic effects to the present study (h^2^ = 0.19)^109^. Future studies could augment this Obsessiveness group factor by adding measures of overt compulsive behaviours, such as the compulsivity scale of the Impulsive–Compulsive Behaviours Checklist, the Obsessive Compulsive Inventory—Revised, or the Dimensional Yale-Brown Obsessive–Compulsive Scale^110^–112.

Our use of a community sample is consistent with the observed quantitative and dimensional organisation of psychiatric phenotypes and with recommendations to study these traits across the full spectrum of severity, which confers increased precision and statistical power for measuring genetic effects^5,6,9^. We have previously demonstrated continuity and normal distribution of the these three phenotypes in a sample combining non-clinical community-based participants and participants with diagnosed obsessive–compulsive disorder and pathological gambling^71^. Individual differences in these phenotypes explained self-reported severity of concurrent psychopathology symptoms, as well as relating to variation in effective connectivity in cortico-thalamo-cortical circuits, whereas diagnostic status did not^71,22^. Nevertheless, it would be informative for future studies to include twins with clinically diagnosed impulse control, obsessive–compulsive, and addiction-related disorders to test the continuity of the additive genetic and environmental factors in explaining clinically-relevant variance in the three phenotypic dimensions identified in this study.

Behavioural genetics studies using a classical twins design require accurate zygosity classification. Genotyping is the gold standard for zygosity classification, but is not always feasible in large-scale twin registries^113^. In these instances, self-reported zygosity is still used and yields high rates of accuracy and agreement with classification by genotype (> 90%)^113–116^. For zygosity classification, we combined responses from both twins on the Peas-in-a-Pod questionnaire, which has demonstrated 93% accuracy in the registry from which our sample was drawn^117^. However, monozygotic twins can be misclassified both pre- and post-natally and incorrectly identify as dizygotic on self-report measures^118^. Thus, our use of the self-report method should be considered a limitation as incorrect zygosity assignment can affect heritability estimates, especially in small samples^119^.

## Conclusions

We provided results from a behavioural genetics study of the phenotypic overlap of impulsivity and compulsivity. The model was constructed using self-report questionnaires capturing psychological processes relevant to impulsivity, intolerance of uncertainty, and obsessive beliefs. Results from univariate twin models revealed that a CE model, representing a modest effect of the common environment, provided the best fit to individual difference variance in the general Impulsive–Compulsive phenotype compared to competing models. Moderate and modest estimates of heritability were obtained for the narrower Impulsivity and Obsessiveness phenotypes. General impulsivity–compulsivity, as operationalised here, may represent an ‘environmental endophenotype’ that indexes a quantitative liability to impulse control, obsessive–compulsive, and substance use disorders, as well as behavioural addictions, associated with exposure to environmental risk factors. The Impulsivity and Obsessiveness dimensions appear to be narrower and partly heritable traits in line with results from previous twin studies.

## Methods

### Participants

The research study methodology was approved by the Monash University Human Research Ethics Committee and Melbourne Human Research Ethics Committee prior to participant recruitment and data collection and proceeded in accordance with the Australian National Statement on Ethical Conduct in Human Research (2007) and the Declaration of Helsinki (1964). Twins were recruited from Twins Research Australia, a large national voluntary registry of 40,000 twin pairs^120^. Seven hundred adult twin pairs (1,400 individual twins) aged 18–55 years were randomly selected from the registry and invited by Twins Research Australia to participate in the study via email. Estimates of response rate and sample size requirements were based on general guidelines for twin modelling research and previous studies^35,40^. Data collection consisted of an online questionnaire battery administered using the Qualtrics Insight Platform [https://monash.az1.qualtrics.com/] over two consecutive sessions of approximately 20 min duration (40 min total). Written informed consent was obtained from each twin participant at the beginning of session one, prior to collecting demographic information and responses to questionnaires. Sample demographic information for the 522 twins that participated (response rate 37.3%), consisting of 195 twin pairs (MZ = 135; DZ = 60) and 132 twin singletons, is provided in Table 3. Zygosity was assigned by combining ratings for both twins on the ‘Two Peas in a Pod’ questionnaire, a method that yields 93% accuracy for zygosity determination and is still widely used and endorsed in the twin modelling literature^114,115,121^. Available data varied across questionnaires due to different response rates across the two sessions and/or missing data: UPPS-P N = 471 [twin 1 n = 230, twin 2 n = 241]; OBQ-44 N = 495 [twin 1 n = 241, twin 2 n = 254]; IUS-12 N = 419 [twin 1 n = 203, twin 2 n = 216]. The total number of twins (including singletons) for which at least partial data was obtained for the subscales scores for use as indicators in the second-order models was N = 487 (302 female), aged 18–55 (M = 39.78, SD = 10.53). Estimates of the three target phenotypes were available for 173 twin pairs (MZ = 118, DZ = 55).

**Table 3:** Demographic variables of twins included in the analyses.

Demographic variables	Twin pairs [N = 195]		Difference test MZ–DZ [t/χ^2^]	Twin singletons [N = 132]	
Zygosity, n	MZ [n = 135]	DZ [n = 60]		MZ [n = 78]	DZ [n = 54]
Age M (SD)	18–55 38.02 (10.37)	19–55 43.77 (9.39)	*t* (388) = − 5.19, p < 0.001 [*d* = 0.58]	18–55 36.28 (10.46)	21–55 42.65 (10.35)
**Sex**					
Sex ratio	F = 174 (64.4%) M = 96 (35.6%)	F = 72 (60.0%) M = 48 (40.0%)	χ(1) = 0.705, *p* = 0.401	F = 37 (47.4%) M = 41 (52.6%)	F = 37 (68.5%) M = 17 (31.5%)
Female/female	86 (63.7%)	25 (41.6%)		38 (48.7%)	22 (40.7%)
Male/male	47 (34.8%)	13 (21.7%)		40 (51.3%)	8 (14.8%)
Female/male	0 (0.0%)	22 (36.70%)		0 (0.0%)	24 (44.4%)
**Parental education**^**a**^					
Started primary school	0 (0.0%)	1			
Completed primary school	4 (1.5%)	0	χ(9) = 7.914, p = 0.543	1 (1.3%)	0 (0.0%)
Started secondary school/high school	30 (11.1%)	8		12 (15.4%)	10 (18.5%)
Completed secondary school/high school	57 (20%)	22		16 (20.5%)	15 (27.8%)
Started vocational/technical school	8 (2.6%)	2		1 (1.3%)	0 (0.0%)
Completed vocational/technical school	52 (19.3%)	28		16 (20.5%)	8 (14.8%)
Started tertiary undergraduate	5 (1.9%)	3		5 (6.4%)	2 (3.7%)
Completed tertiary undergraduate	55 (20.4%)	28		21 (26.9%)	10 (18.5%)
Started tertiary postgraduate	5 (1.9%)	2		0 (0.0%)	0 (0.0%)
Completed tertiary postgraduate	47 (17.8%)	23		6 (7.7%)	9 (16.7%)
Other/missing	7 (0.7%)	3		0 (0.0%)	0 (0.0%)
**Employment**^**a**^					
Unemployed	9 (3.3%)	4 (3.6%)	χ(5) = 3.562, *p* = 0.614	5 (6.4%)	3 (5.6%)
Student	16 (5.9%)	4 (3.6%)		7 (9.0%)	3 (5.6%)
Self-employed	28 (10.4%)	11 (10.0%)		8 (10.3%)	5 (9.3%)
Full time	149 (55.2%)	61 (55.5%)		42 (53.8%)	29 (53.7%)
Part time/casual	46 (17.0%)	28 (25.5%)		12 (15.4%)	11 (20.4%)
Other/missing	22 (8.1%)	12 (10.9%)		4 (5.1%)	3 (5.6%)
**Relationship status**^**a**^				78	54
Married/de facto	163 (60.4%)	79 (71.8%)	χ(5) = 13.013, *p* = 0.023	50 (64.1%)	34 (63.0%)
Partner	29 (10.7%)	8 (7.3%)		6 (7.7%)	3 (5.6%)
Divorced/separated	7 (2.6%)	11 (10.0%)		3 (3.8%)	3 (5.6%)
Single	62 (23.0%)	19 (17.3%)		19 (24.4%)	12 (22.2%)
Widowed	2 (0.7%)	0 (0.0%)		0 (0.0%)	0 (0.0%)
Other/missing	7 (2.6%)	3 (2.7%)		0 (0.0%)	2 (3.7%)

N = 522.

^a^Counts and percentages based on responses of individual twins (MZ = 250, DZ = 110).

### Materials

The 59-item Urgency, Premeditation (lack of), Perseverance (lack of), Sensation Seeking, and Positive Urgency (UPPS-P) Impulsive Behavior Scale measures impulsivity on a 4-point Likert scale (1—‘Agree strongly’ to 4—‘Disagree strongly’) across five dimensions of impulsivity: (1) Negative Urgency—impulsiveness in response to the experience of negative affect; (2) Sensation Seeking—reward-sensitivity/excitement seeking; (3) Lack of Premeditation—a tendency to act without deliberation and forethought; (4) Lack of Perseverance—inability to remain focused and complete a task that is boring or difficult; (5) Positive Urgency—impulsiveness in response to the experience of positive affect^122^. Thirty-nine of the items were recoded such that higher scores on each of the scales represented greater levels of impulsivity in each domain. The Obsessive Beliefs Questionnaire 44-item version (OBQ-44) was used to measure obsessive beliefs across three domains—(1) Importance and Control of Thoughts; (2) Perfectionism and Intolerance of Uncertainty; (3) Responsibility and Threat Estimation) using a 7-point Likert-scale (1—‘Disagree very much to 7—‘Agree very much’)^123^. The Intolerance of Uncertainty Scale 12-item version (IUS-12) is a self-report questionnaire used to measure maladaptive/negative beliefs about uncertainty and its consequences in two related domains—(1) Desire for Predictability and an Active Engagement in Seeking Certainty; (2) Paralysis of Cognition and Action in the Face of Uncertainty—scored using a 5-point Likert scale (1—‘Not at all characteristic of me’ to 5—‘Entirely characteristic of me’)^124^. Several other questionnaires were completed but not analysed in the current study. A list of these questionnaires is provided in Supplementary Information.

### Procedures

The study used a correlational, non-experimental research design. Participants completed the study questionnaires over two sessions. In the first session, the participants completed: (1) a self-reported survey designed ad hoc for this project to collect demographic and clinical data, as well as the OBQ-44. In the second session, participants completed the UPPS-P and IUS-12. Order of administration for the full battery of questionnaires not analysed in this study is provided in Supplementary Information.

### Statistical analyses

#### Analytic strategy

We addressed several limitations of our previous approach to modelling the three phenotypes related to impulsivity and compulsivity^22^. In our previous work, we generated factor score estimates based on separate bifactor models of the UPPS-P, IUS-12, and OBQ-44, which provided the best fit to the data obtained from these scales compared to several competing models. The factor score estimates were then used as indicator variables for the second-order confirmatory factor analysis (CFA) model to derive the three phenotypes. These bifactor models may have provided a better fit to the questionnaire item data compared to the published subscale structure of the UPPS-P, IUS-12, and OBQ-44 simply because they included more parameters and could better account for the observed covariances (i.e. over-parameterisation)^70,87,125^. Additionally, the substantive interpretation of the group factors in bifactor models is difficult and less than adequate reliability of subscale-specific, residual variance is often observed^125^.

We took several steps to address these previous limitations. CFA models were fitted to the UPPS-P, IUS-12, and OBQ-44 self-report data based on their a priori published subscale structure. We compared these results to competing models to evaluate whether the subscales provided a reasonable representation of the latent structure of the data for use as indicators in the higher-order CFA models. Using raw subscales scores as indicators enables the model intercepts and unstandardised loading estimates to be directly and meaningfully compared across studies. This approach facilitates more interpretable solutions and unbiased estimates for the higher-order factor model, whilst also having the added benefit of parsimony. We used a cross-validation approach by fitting the models to data obtained from the first of each twin pair and then replicating the models in the data obtained from the second twin from each pair using invariance testing^126^. This methodology ensured that the models were psychometrically stable and replicable. It also formally tested the assumptions of equivalence of means, variances, and covariances across twin pairs prior to subsequent heritability analyses^35^.

#### Structural equation modeling

Preliminary analyses, including analysis of missing data, non-normality, and outliers, are described in Supplementary Methods online. All structural equation modelling (SEM) was conducted in Mplus 7.31^54^. First-order CFA models of the UPPS-P, IUS-12, and OBQ-44 ordered categorical data were estimated using the weighted least square mean- and variance-adjusted (WLSMV) estimator and theta parameterisation^54^. The WLSMV estimator provides robust parameter estimates and standard errors for non-normally distributed ordered categorical data^88^. Theta parameterisation involves scaling the latent response variables for the questionnaire items by fixing the error variance to one (instead of fixing the latent response variable to one in the alternative delta parameterisation) and is the preferred approach to invariance testing for multi-group comparisons using ordered categorical data^88^. An ‘alternative’ or ‘competing’ models strategy^89,127^ was used to determine the best fitting model in the twin 1 subsample followed by cross-validation in the twin 2 subsample using invariance testing^126^. Higher-order CFA models conducted on the UPPS-P, IUS-12, and OBQ-44 subscale scores for estimation of the dimensional phenotypes used for heritability analyses were estimated using full information maximum likelihood and the Bollen-Stine bootstrap procedure with 10,000 posterior draws to account for missing data and multivariate non-normality^128^. A bifactor model was first estimated in the twin 1 subsample based on the general structure previously reported^22^ and then cross-validated in the twin 2 subsample using invariance testing^126^. Latent variable scaling was performed using the reference variable method, as this is the preferred approach for multi-group comparisons using invariance testing, because it does not assume equality of latent variable variances^88^. Post hoc model fitting was performed by freeing theoretically plausible error covariances for estimation one at a time with reference to modification indices and with adjustment of significance thresholds for multiple comparisons using the Benjamini–Hochberg false discovery rate (B-H FDR; q = 0.05)^50^.

Model fit was assessed using a combination of fit indices, including the chi square test statistic (χ^2^), Root Mean Square Error of Approximation (RMSEA) (ε < 0.05 close approximate fit; ε = 0.05—0.08 close approximate fit; ε = 0.08–1.0 reasonable approximate fit), Comparative Fit Index (CFI) (> 0.90), and Standardised Root Mean Square Residual (SRMR) (< 0.08), or Weighted Root Mean Square Residual (WRMR) for categorical variables^89^. The χ^2^ test statistic is the gold standard metric for evaluating overall model fit and was referred to first. A probability value > 0.05 indicates that the null hypothesis of exact fit of the model reproduced covariance matrix to the observed covariance matrix cannot be rejected^88^. The χ^2^ is overly sensitive to minor model misspecification in datasets characterised by high correlations amongst the observed variables (i.e. conceptually-related items on a questionnaire), when estimated in sample sizes of 200–300 or more^88^. For this reason, we referred to approximate fit indices to adjudicate between the fit of first-order competing models for the UPPS-P, IUS-12, and OBQ-44. Bifactor models often provide a better fit compared to competing models simply because they include more freely estimated parameters^125^. We therefore used information functions based on item response theory analysis to evaluate the reliability of the group factors obtained using alternative model specifications (see Supplementary Figures S1–S10 online)^129^.

Factor score estimates were generated in Mplus using the regression method for the Impulsive–Compulsive, Impulsivity, and Obsessiveness dimensions in the combined sample^54^. The within-trait and cross-trait correlations for the factor score estimates in monozygotic and dizygotic twin pairs are displayed in Table 4. The regression method results in unbiased estimates of individual differences on the underlying latent variable when the factor score estimates are used as exogenous (i.e. independent) variables in a structural equation model^130^. The factor scores estimates were treated as exogenous variables in the variance components models in which they were specified as predictors of the genetic and environment components of the model^35^. We also fixed error variance of the factor score estimates to reflect unreliability of the estimation process based on the factor score determinacies^52^. Heritability analyses were performed in Mplus with variance components models using the maximum likelihood estimator^54^. We estimated statistically nested variance components models for each of the dimensional phenotypes and compared them to determine the best-fitting model^35^. The χ^2^ test statistic and difference test (Δχ^2^) are underpowered to test between competing models with modest sample sizes and few degrees of freedom^88^. We therefore compared competing variance components models using Bayesian conditional posterior probabilities [Pr (Hi | D] to identify the best-fitting model^57^. All direct statistical tests, including z tests, were two-tailed.

**Table 4:** Within-trait and cross-trait correlations for the phenotype factor score estimates in monozygotic and dizygotic twin pairs.

Variable	Age	Impulsive–compulsive_2_	Impulsivity_2_	Obsessiveness_2_
**MZ**				
Age		− 0.196 [− 0.364, − 0.016]	− 0.097 [− 0.273, 0.085]	− 0.125 [− 0.299, 0.057]
Impulsive–compulsive_1_	− 0.232 [− 0.396, − 0.053]	0.365 [0.197,0.512]	− 0.051 [− 0.093, 0.193]	0.134 [− 0.009, 0.272]
Impulsivity_1_	0.037 [− 0.145, 0.216]	0.032 [− 0.112, 0.174]	0.300 [0.126, 0.456]	− 0.016 [− 0.159, 0.127]
Obsessiveness_1_	− 0.047 [− 0.226,0.135]	0.246 [0.107, 0.376]	0.121 [− 0.023, 0.260]	0.224 [0.045,0.389]
**DZ**				
Age		− 0.169 [− 0.416, 0.101]	− 0.129 [− 0.381, 0.141]	− 0.024 [− 0.287, 0.243]
Impulsive–compulsive_1_	− 0.202 [− 0.444, 0.067]	0.367 [0.113, 0.576]	0.041 [− 0.227, 0.303]	0.273 [0.008, 0.502]
Impulsivity_1_	− 0.151 [− 0.400, 0.119]	− 0.032 [− 0.295, 0.235]	− 0.069 [0.328, 0.200]	− 0.041 [− 0.303, 0.227]
Obsessiveness_1_	− 0.006 [− 0.271, 0.260]	0.231 [− 0.037, 0.468]	0.122 [− 0.148, 0.375]	0.137 [− 0.133, 0.388]

95% CI in brackets. MZ N = 118, DZ N = 55. These correlations do not take into account the error of measurement in the factor score estimates due to factor score indeterminacy.

## Supplementary information

Supplementary information.

## Data Availability

The data that support the findings of this study are available from Twins Research Australia [https://www.twins.org.au/] on application and following approval by institutional review board.
