# Supplementary material for: Heritability of overlapping impulsivity and compulsivity dimensional phenotypes
Source: Sci Rep. 2020 Sep 1;10:14378. doi: 10.1038/s41598-020-71013-x (PMC7463011; doi:10.1038/s41598-020-71013-x)
Supplement: Supplementary file 1 — Supplementary information. [file 41598_2020_71013_MOESM1_ESM.docx]

**Heritability of overlapping impulsivity and compulsivity dimensional phenotypes**

Jeggan Tiego, Samuel R. Chamberlain, Ben J. Harrison, Andrew Dawson, Lucy Albertella, George J. Youssef, Leonardo F. Fontenelle, Murat Yücel

**SUPPLEMENTARY INFORMATION**

July 31^st^, 2020

Supplementary methods word count: 676

Supplementary results word count: 835

Number of tables: 11

Number of figures: 13

**SUPPLEMENTARY METHODS**

**Materials**

Several other questionnaires were completed, but were not analysed in the current study. These were the Impulsive-Compulsive Behavior Checklist (ICBC) ^1^; Questionnaire for Impulsive-Compulsive Disorders in Parkinson's Disease; Barratt Impulsivity Scales - Eleventh Edition (BIS-11) and Grattan Compulsivity Scale (GCS) ^2^; Life Events Questionnaire (LES); Obsessive-Compulsive Inventory-Revised (OCI-R) ^3^; Problem Gambling Severity Index (PGSI) ^4,5^; Behavioural Inhibition/Activation Scales (BIS/BAS) ^6^; Childhood Trauma Questionnaire (CTQ) ^7^; and Quality of Life Enjoyment and Satisfaction Questionnaire – Short Form ^8,9^(QLESQ-SF).

**Procedures**

Participants completed the study questionnaires over two sessions. In the first session, the participants completed: 1) a self-reported survey designed *ad hoc* for this project to collect demographic and clinical data; as well as the 2) ICBC; 3) Questionnaire for Impulsive-Compulsive Disorders in Parkinson's Disease; 4) BIS-11 and GCS; 5) OBQ-44; and 6) LEQ. In the second session, participants completed the: 7) OCI-R; 8) PGSI; 9) UPPS-P ;10) IUS-12; 11) BIS/BAS; 12) CTQ; and the 13) QLESQ-SF.

**Statistical Analyses**

**Missing data analysis, normality, and outliers.**

Data screening and preliminary analyses were conducted in SPSS 23. All available data were used for the first-order CFAs to obtain the most stable and precise estimates possible. Data were analysed separately in two subsamples comprising twin 1 and twin 2 of each pair. Available data were included in the first-order CFAs even if one of the twin pair did not complete the study. Summed raw subscales scores were calculated from available item data and were coded as missing for participants that failed to respond to one or more items contributing to that scale (proportion of missingness ranged from *n* = 2 [0.4%] for Responsibility and Threat-Estimation to n = 82 [16.8%] for Paralysis of Cognition and Action in the Face of Uncertainty, see supplementary material for more information). Missing data at the level of the questionnaire subscales are summarised in Table S1. At least partial data were available for 487 twins (including singletons) for estimation of the second-order CFA model. Missingness at the subscale level was assessed using Little’s (1988) missing completely at random (MCAR) test^10,11^. Missing data analysis indicated that phenotypic data for the questionnaire subscales were not MCAR for the total sample (χ^2^(122) = 163.664, *p* = .007), but the data were MCAR (χ^2^(94) = 113.776, *p* = .081) once twin singletons and/or twins with unknown zygosity (*n* = 141) not used in the final heritability analyses were removed.

Univariate outliers for the nine subscale scores were independently identified and removed from the twin 1 and twin 2 subsamples using a sequential fence procedure constructed using the upper and lower quartiles, defined as: *f*_Q_ = n/4 + (1/4), and a 2.2 multiplicative of the interquartile range ^12^. This resulted in removal of three cases for Paralysis of Cognition and Action in the Face of Uncertainty from the twin 1 subsample, as well as one case removed for Lack of Perseverance and Importance and Control of Thoughts. In the twin 2 subsample, one case was identified and removed from the Lack of Premeditation and Paralysis of Cognition and Action in the Face of Uncertainty subscales. Only one case exceeding the critical Mahalanobis distance [χ^2^(9) = 27.877, *p* < .001] was identified and removed from the twin 2 subsample ^13^. Tests of multivariate normality were conducted using the SPSS macro provided by DeCarlo (1997). An omnibus test of multivariate normality, based on Small’s (1980) statistics was significant for both the twin 1 (*χ*^2^(18) = 87.879, *p* <.001), and twin 2 (*χ*^2^(18) =91.210, *p* <.001) subsamples, indicating that the data set violated the assumption of multivariate normality ^14,15^. Following cross-validation of the second-order model using invariance testing, the subsamples were combined and re-screened for outliers and normality. Cases were removed from Lack of Premeditation (*n* = 6), Lack of Perseverance (*n* = 2), Paralysis of Cognition and Action in the Face of Uncertainty (*n* = 8), and Importance and Control of Thoughts (*n* = 1). Only one case exceeded the critical Mahalanobis distance [χ^2^(9) > 27.877, *p* < .001] and was removed. The combined sample was multivariate non-normal (*χ*^2^(18) = 166.248, *p* <.001).

**SUPPLEMENTARY RESULTS**

**Descriptive Statistics**

Descriptive statistics for the UPPS-P, IUS-12, and OBQ-44 subscales are summarised in Table S2. Subscale means were broadly comparable to those reported in the previous study conducted in a large online sample ^16^. Exceptions were the lower mean scores observed in the twin sample for Desire for Predictability and an Active Engagement in Seeking Certainty subscales of the IUS-12, and the Perfectionism, and Responsibility and Threat Estimation subscales of the OBQ-44 along with an overall truncation of range revealed by smaller subscale standard deviations. These differences likely reflected properties of the smaller and more homogenous twin sample compared to the online sample. Internal consistency reliability was generally good to excellent for the questionnaire subscales (α =.83 - .93) except for the Lack of Perseverance subscale from the UPPS-P (α =.50), which was below acceptable standards of reliability for use of the observed raw subscale scores in subsequent analyses ^17^. However, the use of SEM circumvents this problem with error variance (i.e. subscale variance not covarying with other subscales in the model) excluded from the higher-order dimensional phenotypes model.

Table S1

*Proportion of Missingness for Subscale Scores Used as Indicator Variables in the Second-Order Confirmatory Factor Analysis Model*

| Subscale | N | Missing | |
| --- | --- | --- | --- |
|  |  | N | % |
| Lack of Premeditation | 456 | 31 | 6.4 |
| Urgency | 454 | 33 | 6.8 |
| Sensation Seeking | 456 | 31 | 6.4 |
| Lack of Perseverance | 458 | 29 | 6.0 |
| Desire for Predictability and an Active Engagement in Seeking Certainty | 409 | 78 | 16.0 |
| Paralysis of Though and Action in the Face of Uncertainty | 405 | 82 | 16.8 |
| Responsibility and Threat-Estimation | 485 | 2 | 0.4 |
| Perfectionism | 484 | 3 | 0.6 |
| Importance and Control of Thoughts | 481 | 6 | 1.2 |

*Note. N* = 487

Table S2

*Descriptive Statistics for the UPPS-P, IUS-12, and OBQ-44 Scales in the Twin Sample*

| Variable  Variable | *M* | 95% *CI*  *LL UL* | | *SD* | *Range* | *Skewness* | *Kurtosis* | *Reliability*^1^ |
| --- | --- | --- | --- | --- | --- | --- | --- | --- |
| UPPS-P |  |  |  |  |  |  |  |  |
| PosUrge | 24.47 | 23.90 | 25.05 | 6.33 | 14 - 42 | .420 | -3.06^**^ | .93 |
| NegUrge | 25.71 | 25.18 | 26.23 | 5.77 | 12 - 43 | .959 | .473 | .83 |
| Premed | 22.73 | 22.37 | 23.09 | 3.95 | 12 - 33 | -1.42 | 1.70 | .83 |
| Persev | 20.28 | 19.92 | 20.64 | 3.92 | 10 - 31 | -.674 | .134 | .50 |
| SensSeek | 29.24 | 28.62 | 29.86 | 6.69 | 13 - 48 | .670 | -.580 | .87 |
| IUS |  |  |  |  |  |  |  |  |
| Pred | 16.49 | 15.95 | 17.04 | 5.64 | 7 - 35 | 4.76^***^ | -.804 | .89 |
| Para | 8.12 | 7.73 | 8.51 | 3.99 | 5 - 24 | 12.16^***^ | 7.10^***^ | .93 |
| OBQ-44 |  |  |  |  |  |  |  |  |
| ICT | 27.30 | 26.27 | 28.32 | 11.41 | 12 - 69 | 7.49^***^ | 1.17 | .87 |
| RT | 48.91 | 47.32 | 50.50 | 17.82 | 16 - 108 | 3.19^**^ | -.875 | .91 |
| PC | 56.31 | 54.56 | 58.06 | 19.62 | 18 - 112 | 2.44^*^ | -1.71 | .92 |

*Note: N* = 487*. M =* Mean; CI = Confidence Interval; *LL* – Lower Limit; *UL* = Upper Limit; *SD* = Standard Deviation; *n* = size of subsample.

UPPS = UPPS - P Impulsive Behavior Scale; PosUrge = Positive Urgency; NegUrge = Negative Urgency; Premed = Lack of Premeditation; ; Persev = Lack of Perseverance; SensSeek = Sensation Seeking; IUS-12 = Intolerance of Uncertainty – 12-item version; Pred = Desire for Predictability and an Active Engagement in Seeking Certainty; Para = Paralysis of Cognition and Action in the Face of Uncertainty; OBQ-44 = Obsessive Beliefs Questionnaire – 44-item version; ICT = Importance and Control of Thoughts; RT = Responsibility and Threat Estimation; PC = Perfectionism.

^1^ Internal consistency reliability calculated as Cronbach’s alpha (α).

*** *p* < .001 ** *p* < .01. * *p* < .05.

**First-Order CFA Models**

**CFA models in twin 1 subsample.**

Competing models, including bifactor models and the published subscale structure and one-factor models, were estimated for the UPPS-P, IUS-12, and OBQ-44. Fit statistics for these models are provided in tables S3 – S5. As expected, bifactor models provided statistically superior fit to the data compared to the *a priori* subscale structure for each of the three questionnaires. The information functions generated for the subscales in the correlated factors models and corresponding group factors obtained from bifactor modelling were very similar in shape (see *Figures S1- S7* supplementary material) for the UPPS-P and IUS-12. Reliability of measurement (*r_xx_* = 1 – 1/*I*) ^18^ increased for Negative Urgency (*Figure S2*) and Lack of Perseverance (*Figure S4*) when measured as group factors in the bifactor model compared to subscales in the correlated five-factor model, and measurement reliability for Sensation Seeking (*Figure S3*) was comparable in both models. Measurement reliability was also comparable for Paralysis of Cognition and Action in the Face of Uncertainty when modeled as a subscale or group factor (*Figure S7*). However, in all other cases there was loss of information over the full distribution of the latent trait (θ) for the remaining measured constructs when measured as group factors in a bifactor model. The loss of information was most marked for the OBQ-44 subscales [e.g. *I* = 13.69 (*r_xx_* = .93) to *I* = 4.75 (*r_xx_* = .79) at mean levels of Importance and Control of Thoughts; from *I* = 55.93 (*r_xx_* = .98) to *I* = 3.61 (*r_xx_* = .72) at mean levels of Responsibility and Threat Estimation; and from *I* = 29.44 (*r_xx_* = .97) to *I* ~ 9 (*r_xx_* = .89) at mean levels of Perfectionism] and tended to be greater at the extreme ends of the distribution of the latent traits (e.g. + 2 *SD*).

These results indicated that the bifactor models introduced substantially more complexity in the parameterisation and interpretability of these constructs without a general improvement in measurement precision. For these reasons, along with those discussed in the Methods section, model solutions that preserved the *a priori* factor structure of the UPPS-P, IUS-12, and OBQ-44 were preferred over the bifactor models. The only exception was that a four-factor model that constrained the factor intercorrelation between Negative Urgency and Positive Urgency to one provided a more parsimonious fit to the UPPS-P data than the five-factor model based on the published subscale structure (χ^2^(1641) = 2682.033, *p* < .001, RMSEA = .053 [90%*CI* = .049, .056]; CFI = .906, WRMR = 1.504, Δχ^2^(1) = .641, Δ*p* = .428).

**Cross-validation of CFA models in the twin 2 subsample.**

The first-order CFA models were cross-validated in the twin 2 subsample using invariance testing. Results of invariance testing are displayed in Table S6. The two-factor IUS-12 and three-factor OBQ-44 models demonstrated full measurement invariance along with equivalence of the factor variances, and equivalence of factor covariances for the OBQ-44. The UPPS-P exhibited partial measurement invariance with minor differences observed in two item loadings and the threshold parameters of six additional items distributed across the five factors. These minor differences in item parameters are unlikely to translate to meaningful differences in the measurement and interpretation of the phenotypes between groups ^19-21^. Only one parameter for each model was freely estimated to obtain partial equality of factor variances and covariances for the UPPS-P five-factor model in the twin 2 subsample. Equality of means between the twin 1 and twin 2 subsamples was also demonstrated for each of the UPPS-P, IUS-12, and OBQ-44 subscales (see Table S7). A four-factor model of the UPPS-P with the intercorrelation between Negative Urgency and Positive Urgency constrained to one also provided a more parsimonious fit to the data than the five-factor model in the twin 2 subsample (χ^2^(1638) = 2922.869, *p* < .001, RMSEA = .057 [90%*CI* = .054, .060; CFI = .902, WRMR = 1.550, Δχ^2^(1) = .003, Δ*p* = .956).

Table S3

*Summary of Fit Statistics for the Different Competing Confirmatory Factor Analysis Models for the UPPS-P in the Twin 1 Subsample*

|  | Model | *df* | *χ*^2^ | *p* | RMSEA (90%*CI*) | CFI | WRMR |
| --- | --- | --- | --- | --- | --- | --- | --- |
| 1 | Bi-Factor ^1^ | 1591 | 2566.402 | <.001 | .052 (.048 - .055) | .912 | 1.390 |
| 2 | Five-Factor ^1,2^ | 1640 | 2687.025 | <.001 | .053 (.049 - .056) | .905 | 1.503 |
| 3 | Four Factor 1^3^ | 1641 | 2682.033 | <.001 | .053 (.049 - .056) | .906 | 1.504 |
| 4 | Four Factor 2 | 1652 | 6970.678 | <.001 | .115 (.112 - .118) | .763 | 2.927 |
| 5 | One Factor | 1652 | 5171.940 | <.001 | .096 (.093 - .099) | .681 | 2.509 |

*Note.* UPPS-P = UPPS-P Impulsive Behavior Scale + Positive Urgency Measure. Thirty-nine items were recoded so that higher scores on each of the factors represent greater levels of impulsivity. *df* = Degress of Freedom; *χ*^2^ = Chi square value for test of model fit estimated using the Weighted Least Square Mean- and Variance-Adjusted estimator (WLSMV); *p =* significance value of the chi square test statistic; RMSEA = Root Mean Square Error of Approximation; CI = Confidence Interval; WRMR = Weighted Root Mean Square Residual; CFI = Comparative Fit Index. Δ*df* = delta degrees of freedom. Δ*χ*^2^ = delta chi square. Δ*p* = significance value of the delta chi square test statistic. *n* = 230.

^1^ Included error covariance terms; ^2^ Nested with respect to the bifactor model. ^3^ Factor covariance between Negative and Positive Urgency constrained to one.

Table S4

*Summary of Fit Statistics for the Different Competing Confirmatory Factor Analysis Models for the Intolerance of Uncertainty Scale (IUS-12) in the Twin 1 Subsample*

|  | Model | *df* | *χ*^2^ | *p* | RMSEA (90%*CI*) | CFI | WRMR | Δ*df* | Δ*χ*^2^ | Δ*p* |
| --- | --- | --- | --- | --- | --- | --- | --- | --- | --- | --- |
| 1 | Bi-Factor ^1^ | 38 | 51.695 | .068 | .042 (.000 - .069) | .996 | .411 |  |  |  |
| 2 | Two-Factor ^1,2,3^ | 48 | 98.801 | <.001 | .072 (.052 - .092) | .985 | .710 | 10 | 41.255 | <.001 |
| 3 | One-Factor ^1,2,3^ | 49 | 275.857 | <.001 | .151 (.134 - .169) | .932 | 1.383 | 11 | 150.385 | <.001 |

*Note.* IUS = Intolerance of Uncertainty Scale – 12-item version. *df* = Degress of Freedom; *χ*^2^ = Chi square value for test of model fit estimated using the Weighted Least Square Mean- and Variance-Adjusted estimator (WLSMV); *p =* significance value of the chi square test statistic; RMSEA = Root Mean Square Error of Approximation; *CI* = Confidence Interval; WRMR = Weighted Root Mean Square Residual; CFI = Comparative Fit Index. Δ*df* = delta degrees of freedom. Δ*χ*^2^ = delta chi square. Δ*p* = significance value of the delta chi square test statistic. *n* = 203

^1^ Included error covariances.

^2^ Nested with respect to the bifactor model.

^3^ Models taken from Birrell et al. (2011).

Table S5

*Summary of Fit Statistics for the Different Competing Confirmatory Factor Analysis Models for the OBQ-44 in the Twin 1 Subsample*

|  | Model | *df* | *χ*^2^ | *p* | RMSEA (90%*CI*) | CFI | WRMR | Δ*df* | Δ*χ*^2^ | Δ*p* |
| --- | --- | --- | --- | --- | --- | --- | --- | --- | --- | --- |
| 1 | Bi-Factor ^1,2^ | 855 | 1532.019 | <.001 | .057 (.053 - .062) | .939 | 1.065 |  |  |  |
| 2 | Three-Factor ^1,3^ | 884 | 1677.550 | <.001 | .061 (.057 - .065) | .928 | 1.183 | 29 | 186.157 | <.001 |
| 3 | One-Factor ^1,4^ | 887 | 1787.875 | <.001 | .065 (.061 - .069) | .919 | 1.299 | 3 | 33.658 | <.001 |
| 4 | Four-Factor ^1,5^ | 881 | 1829.381 | <.001 | .067 (.063 - .071) | .914 | 1.251 |  |  |  |

*Note.* OBQ-44 = Obsessive Beliefs Questionnaire – 44-item version. *df* = Degress of Freedom; *χ*^2^ = Chi square value for test of model fit estimated using the Weighted Least Square Mean- and Variance-Adjusted estimator (WLSMV); *p =* significance value of the chi square test statistic; RMSEA = Root Mean Square Error of Approximation; CI = Confidence Interval; WRMR = Weighted Root Mean Square Residual; CFI = Comparative Fit Index; Δ*df* = delta degrees of freedom. Δ*χ*^2^ = delta chi square. Δ*p* = significance value of the delta chi square test statistic. *n* = 241.

^1^ Included error covariances. ^2^ Bi-Factor model included three orthogonal group factors – Perfectionism-Specific, Importance and Control of Thought-Specific, and Responsibility and Threat Estimation-Specific ^22^. ^3^ Nested with respect to the bifactor model – three-factor model included: 1) Perfectionism, 2) Importance and Control of Thoughts, and 3) Responsibility and Threat Estimation ^22^. ^4^ Nested with respect to the three-factor model - factor covariances constrained to one. ^5^ Four-factor model included: 1) Perfectionism and Intolerance of Uncertainty, 2) Importance and Control of Thoughts, 3) Responsibility, and 4) Overestimation of Threat ^23^.

A) B)


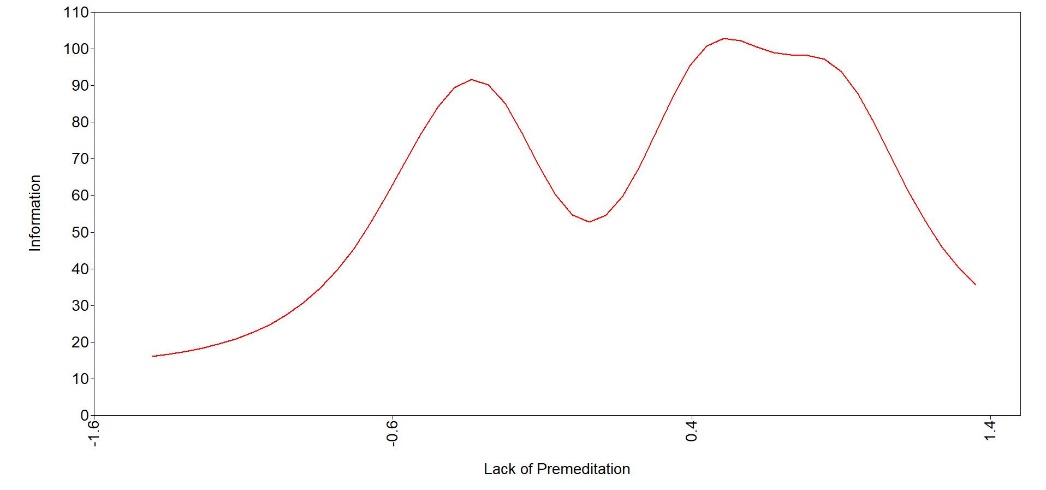

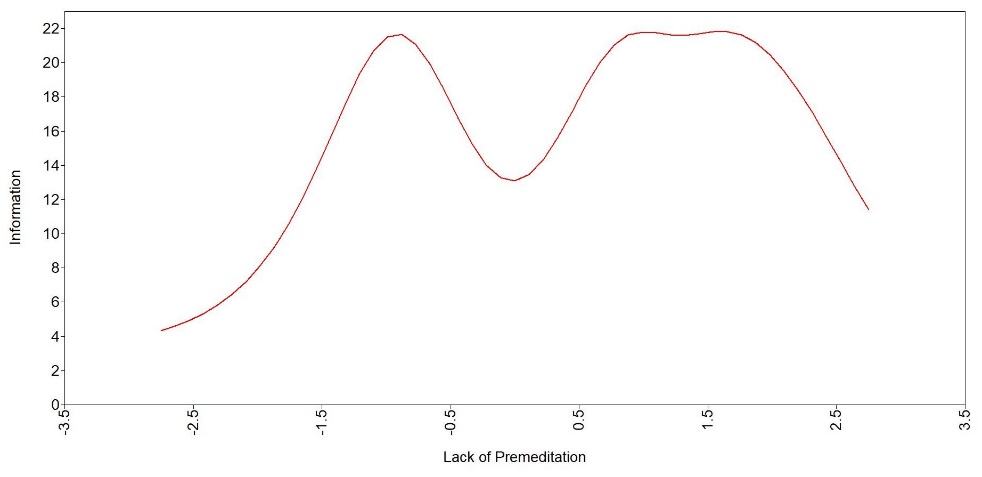


*Figure S1.* Total information functions for UPPS-P Lack of Premeditation A) subscale in the five-factor model; and B) group factor in the bifactor model.

A) B)


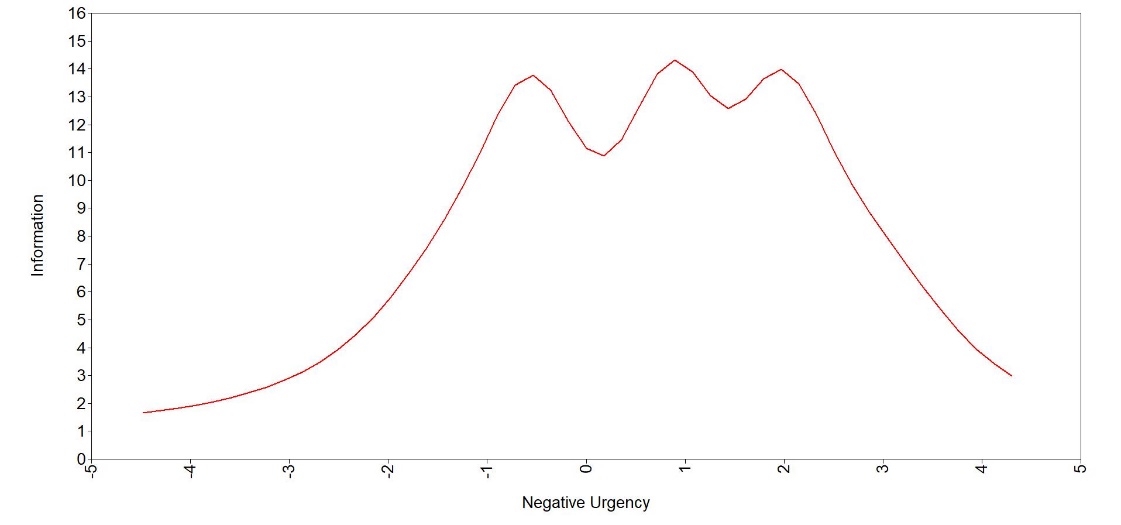

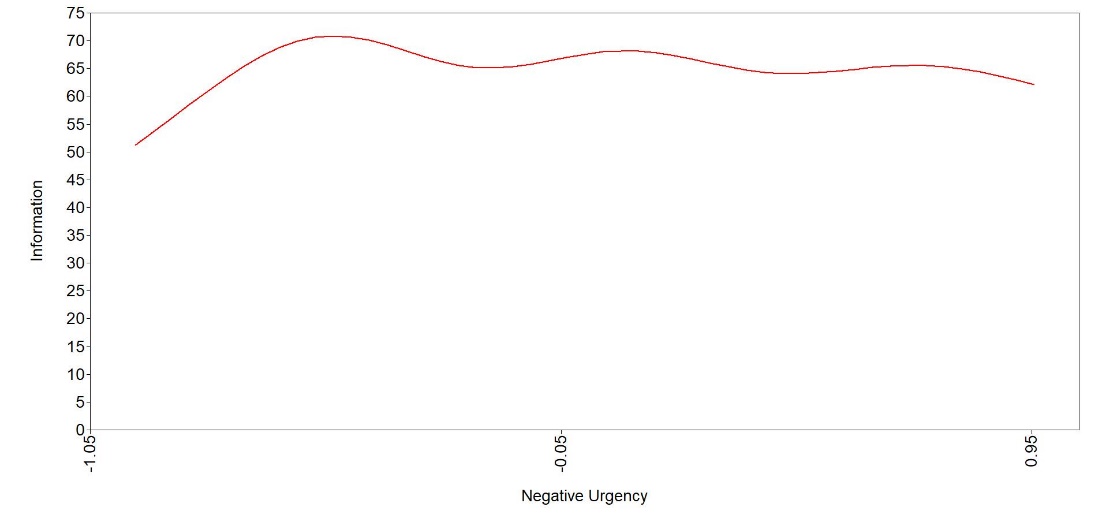


*Figure S2.* Total information functions for UPPS-P Negative Urgency A) subscale in the five-factor model; and B) group factor in the bifactor model.

A) B)


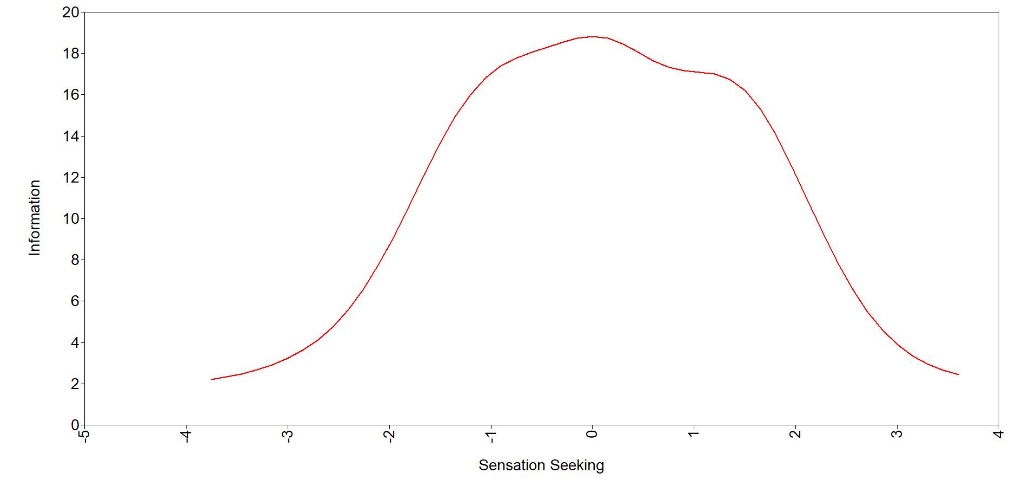

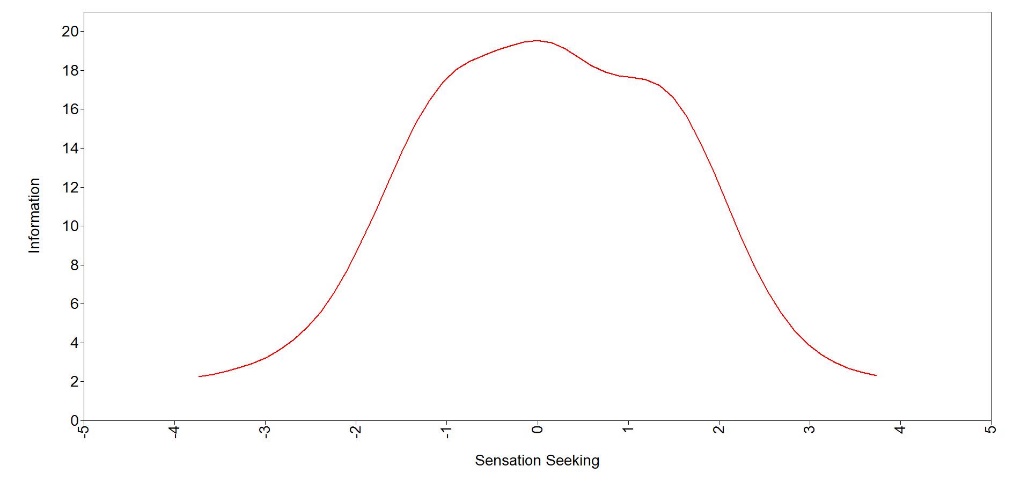


*Figure S3.* Total information functions for UPPS-P Sensation Seeking A) subscale in the five-factor model; and B) group factor in the bifactor model.

A) B)


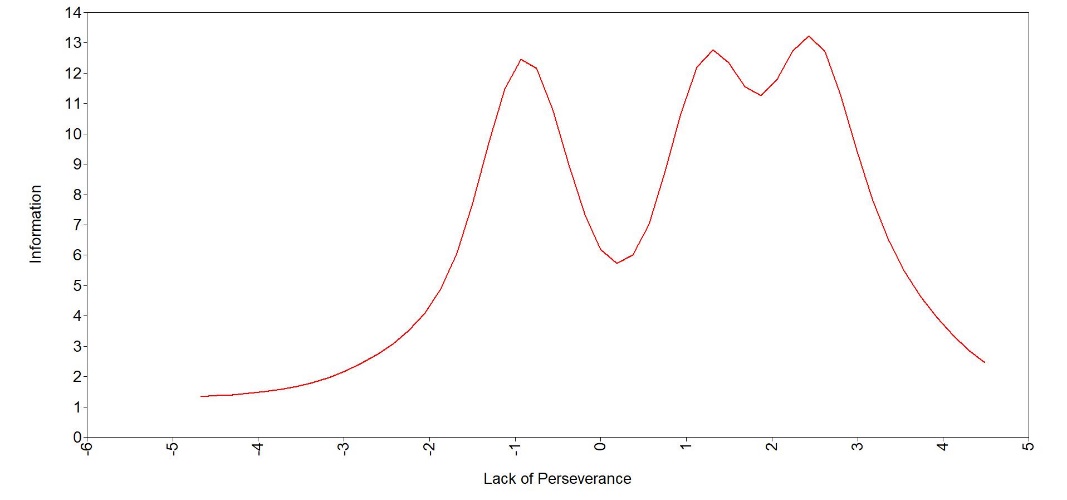

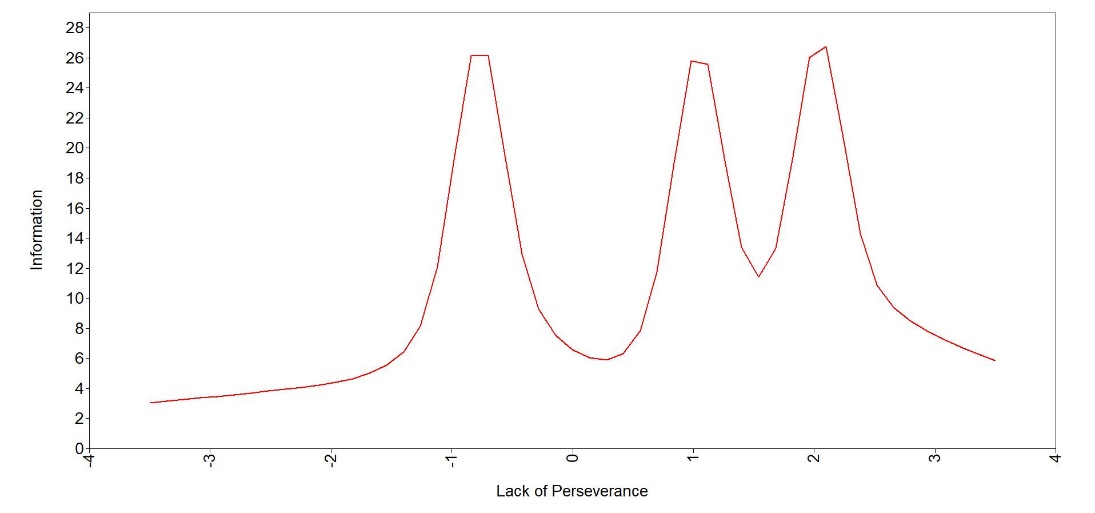


*Figure S4.* Total information functions for UPPS-P Lack of Perseverance A) subscale in the five-factor model; and B) group factor in the bifactor model.

A) B)


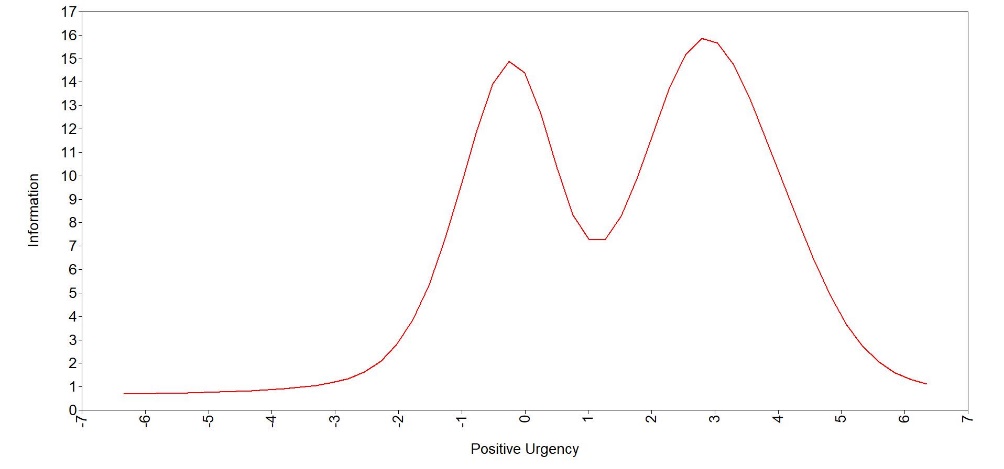

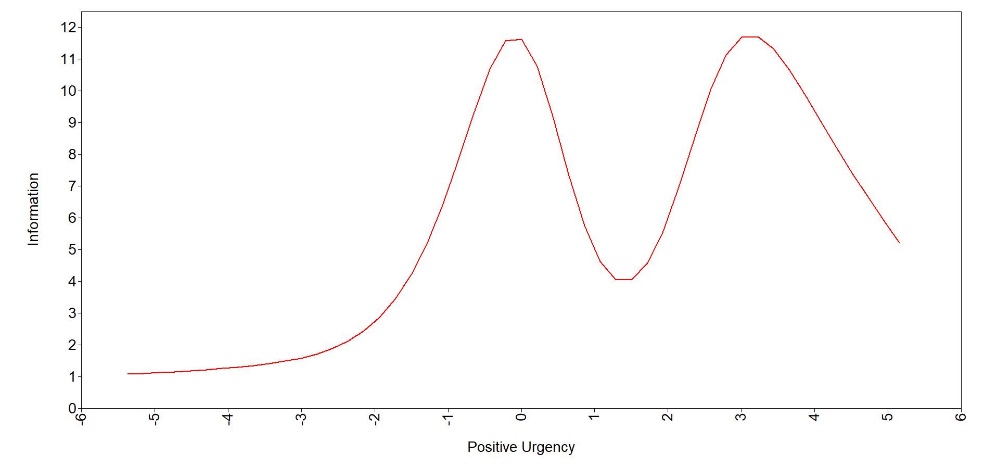


*Figure S5.* Total information functions for UPPS-P Positive Urgency A) subscale in the five-factor model; and B) group factor in the bifactor model.

A) B)


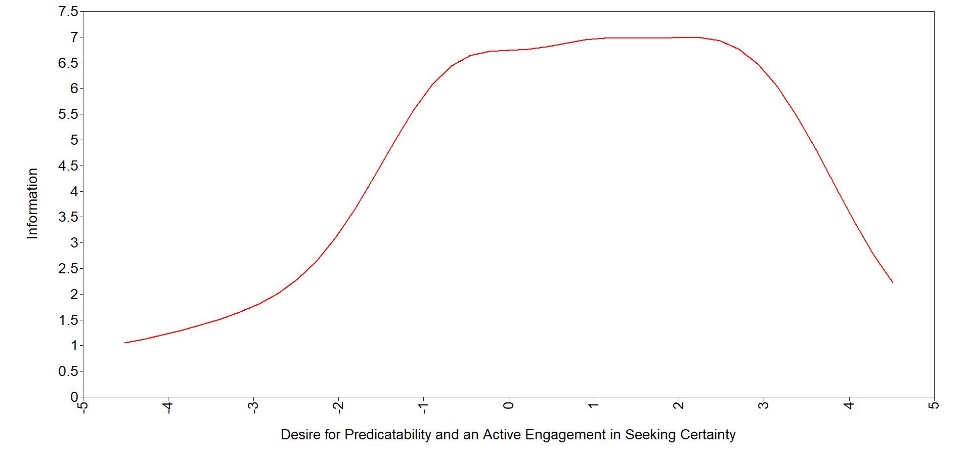

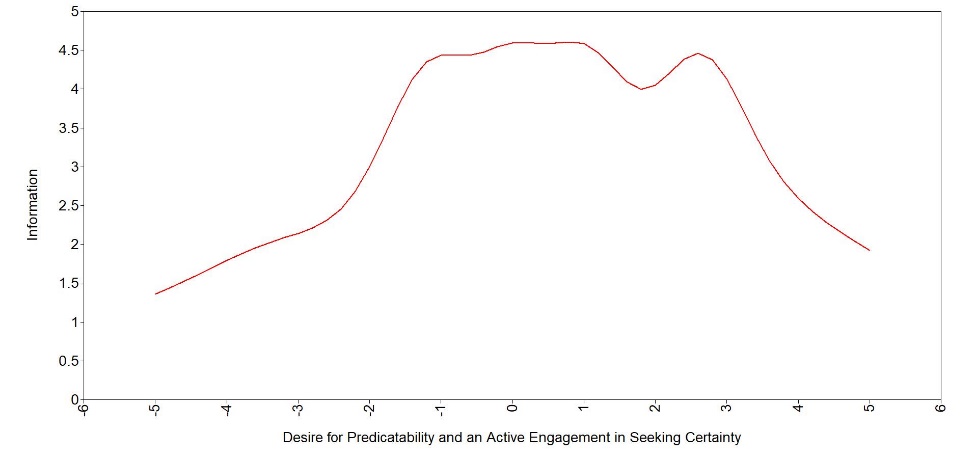


*Figure S6.* Total information functions for IUS-12 Desire for Predictability and an Active Engagement in Seeking Certainty A) subscale in the two-factor model; and B) group factor in the bifactor model.

A) B)


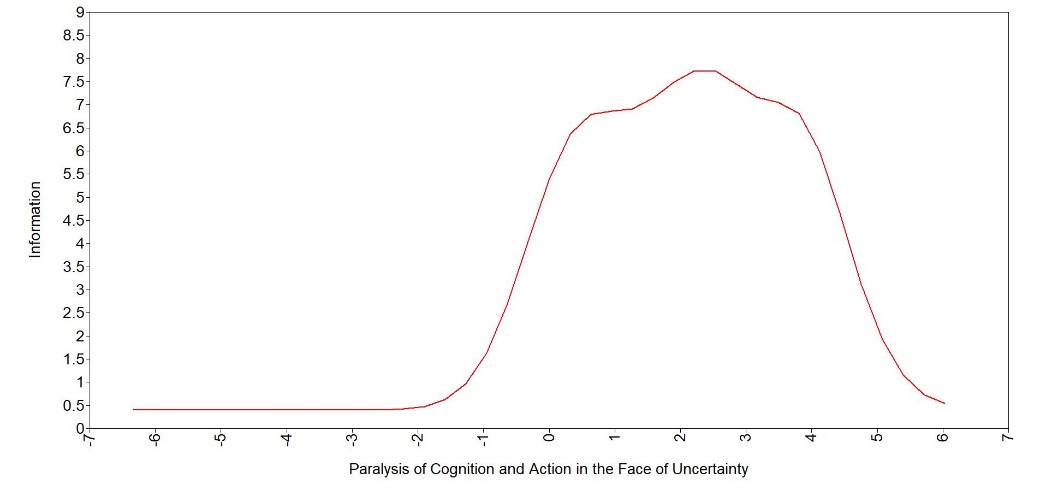

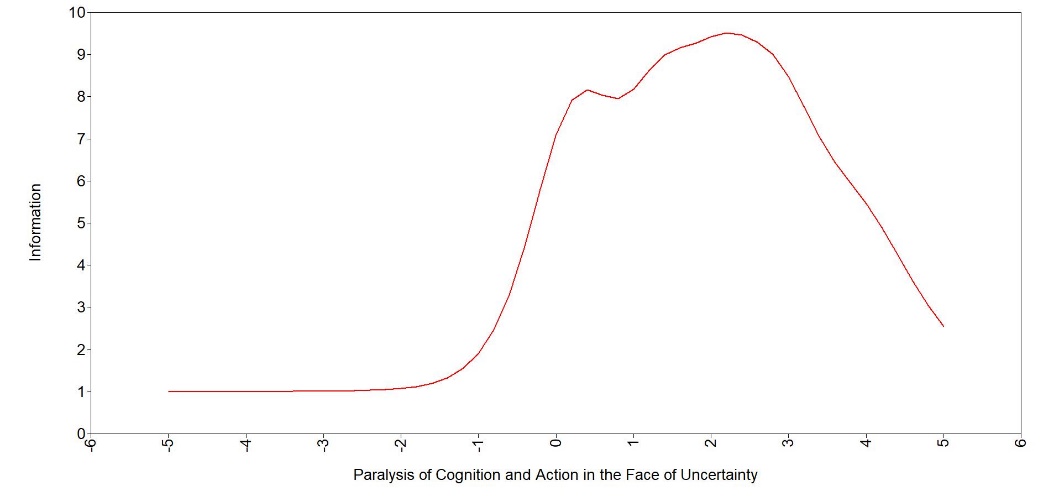


*Figure S7.* Total information functions for IUS-12 Paralysis of Cognition and Action in the Face of Uncertainty A) subscale in the two-factor model; and B) group factor in the bifactor model.

A) B)


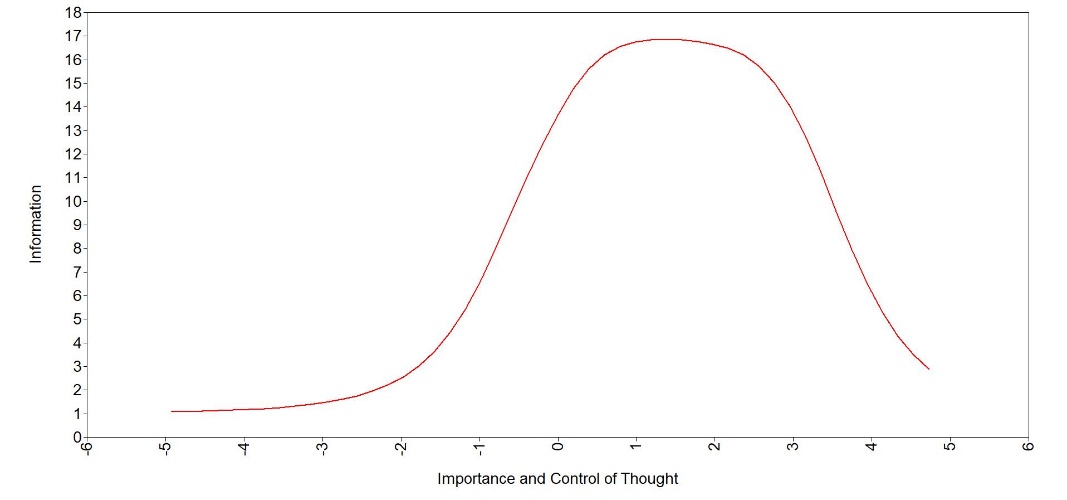

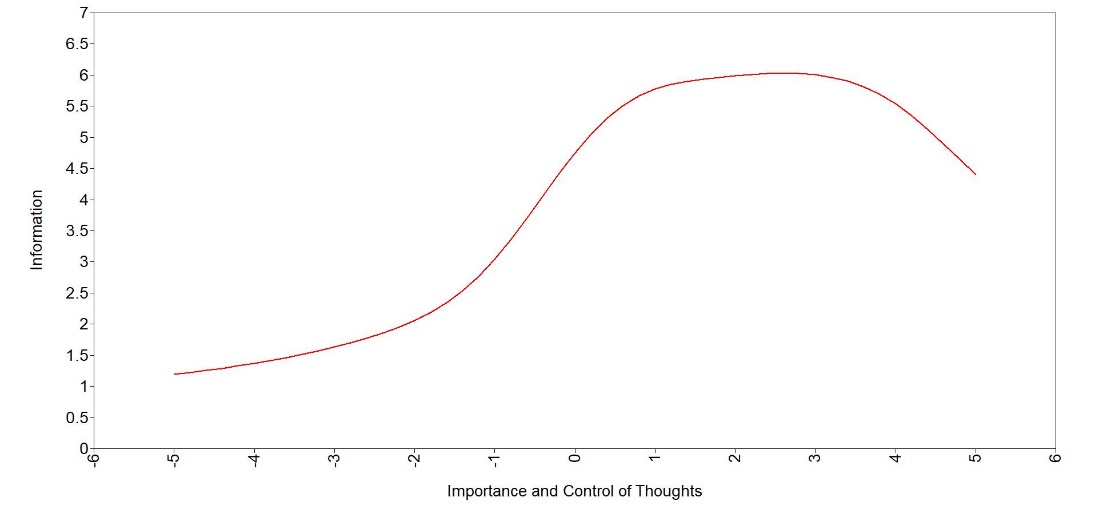


*Figure S8.* Total information functions for OBQ-44 Importance and Control of Thoughts A) subscale in the three-factor model; and B) group factor in the bifactor model.

A) B)


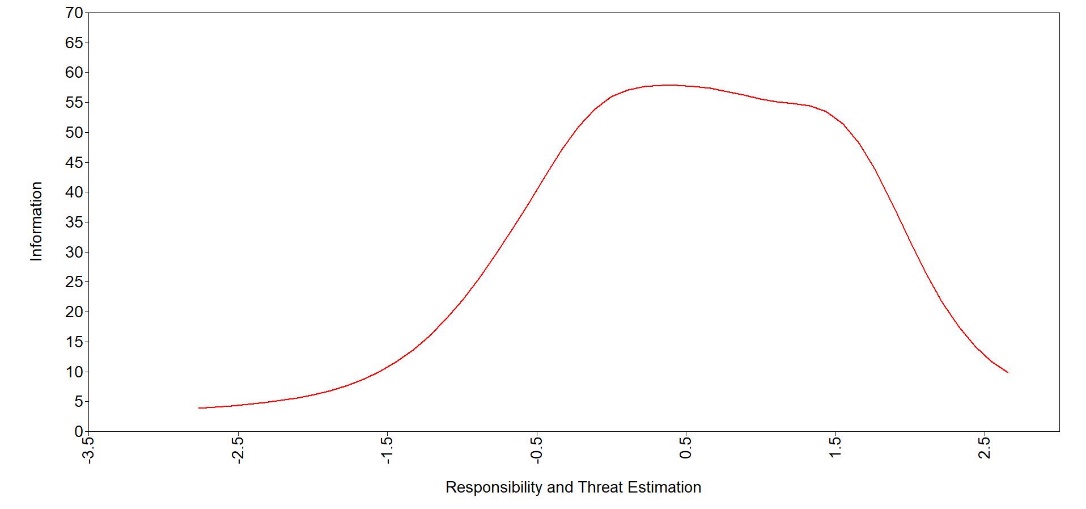

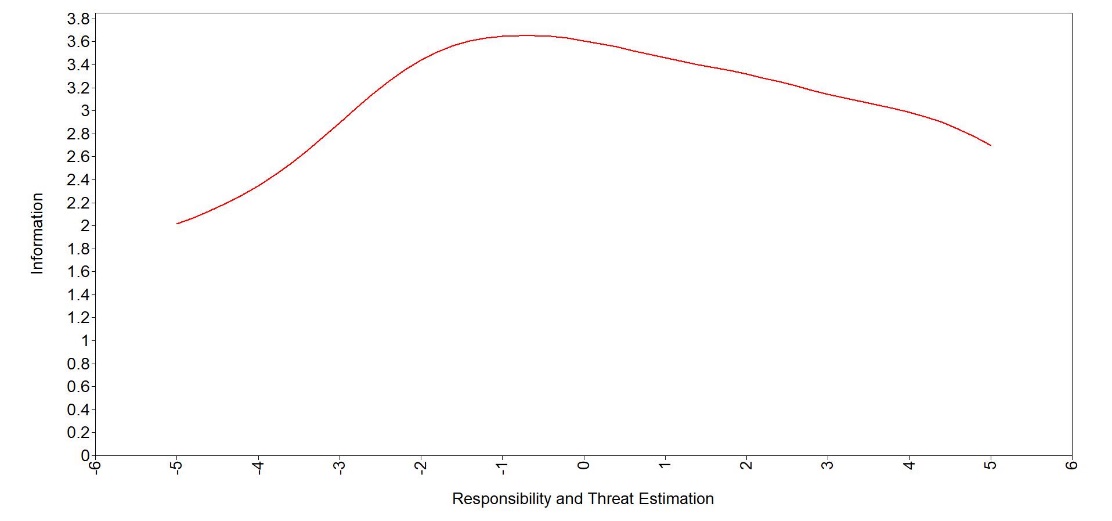


*Figure S9.* Total information functions for OBQ-44 Responsibility and Threat Estimation A) subscale in the three-factor model; and B) group factor in the bifactor model.

A) B)


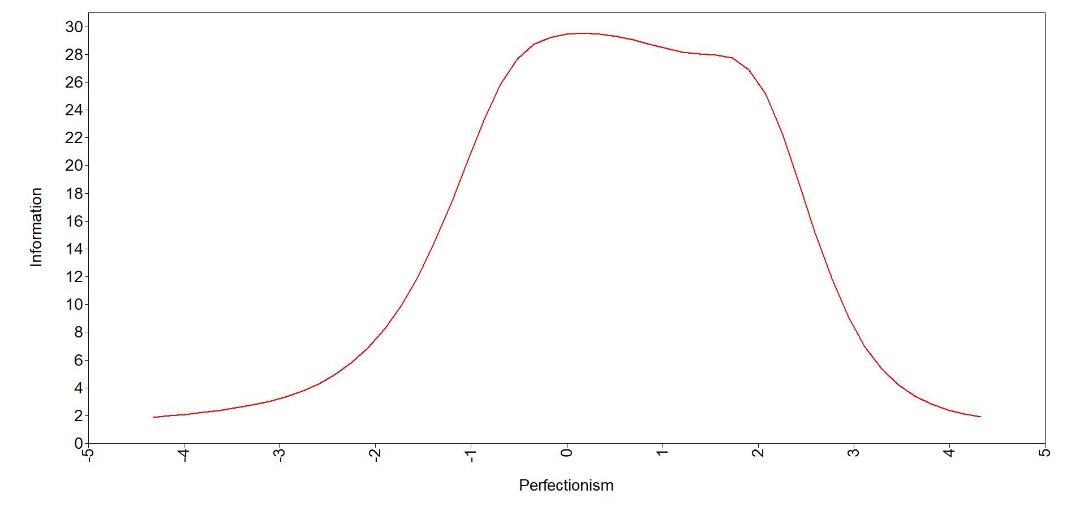

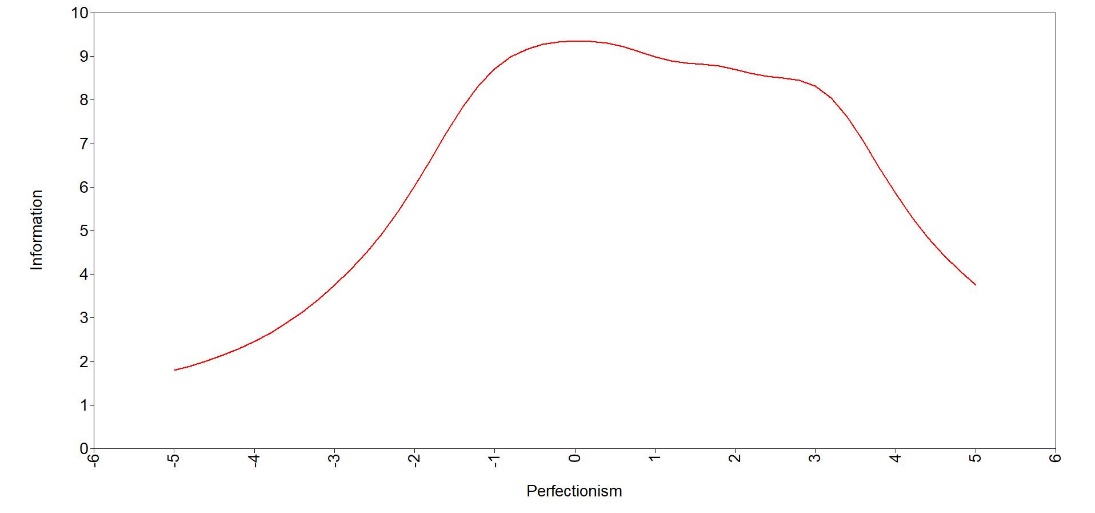


*Figure S10.* Total information functions for OBQ-44 Perfectionism A) subscale in the three-factor model; and B) group factor in the bifactor model.

Table S6

*Results of Invariance Testing for the UPPS-P, IUS-12, and OBQ-44 in the Calibration and Validation Subsamples*

|  | Model | *df* | *χ*^2^ | *p* | RMSEA (90%*CI*) | CFI | WRMR | Δ*df* | Δ*χ*^2^ | Δ*p* |
| --- | --- | --- | --- | --- | --- | --- | --- | --- | --- | --- |
| UPPS-P | Configural^1^ | 3295 | 5684.383 | <.001 | .055 (.053 - .058) | .901 | 2.180 |  |  |  |
|  | Measurement^2^ | 3497 | 5583.922 | <.001 | .050 (.048 - .053) | .913 | 2.210 | 206 | 235.215 | .079 |
|  | Factor Variances^3^ | 3501 | 5518.427 | <.001 | .049 (.047 - .052) | .916 | 2.219 | 4 | 4.818 | .307 |
|  | Factor Covariances^4^ | 3513 | 5182.551 | <.001 | .045 (.042 - .047) | .931 | 2.281 | 12 | 19.230 | .083 |
| IUS-12 | Configural | 101 | 170.559 | <.001 | .057 (.042 - .072) | .993 | .923 |  |  |  |
|  | Measurement | 151 | 214.126 | .001 | .045 (.030 - .058) | .994 | 1.233 | 50 | 67.167 | .053 |
|  | Factor Variances | 153 | 204.973 | .003 | .040 (.024 - .054) | .995 | 1.345 | 2 | 3.399 | .183 |
|  | Factor Covariance | 154 | 211.757 | .001 | .042 (.027 - .056) | .994 | 1.513 | 1 | 4.233 | .040 |
| OBQ-44 | Configural | 1791 | 3549.299 | <.001 | .063 (.060 - .066) | .925 | 1.745 |  |  |  |
|  | Measurement | 2074 | 3197.148 | <.001 | .047 (.044 -.050) | .952 | 1.869 | 283 | 294.231 | .311 |
|  | Factor Variances | 2077 | 2977.975 | <.001 | .042 (.038 -.045) | .961 | 1.870 | 3 | .769 | .857 |
|  | Factor Covariances | 2080 | 2618.659 | <.001 | .032 (.028 -.036) | .977 | 1.874 | 3 | 1.265 | .738 |

*Note.* UPPS-P = UPPS Impulsive Behavior Scale + Positive Urgency Measure (*N* = 471, twin 1 *n* = 230, twin 2 *n* = 241). OBQ-44 = Obsessive Beliefs Questionnaire – 44-item version (*N* = 495, twin 1 *n* = 241, twin 2 *n* = 254). IUS -12 = Intolerance of Uncertainty – 12-item version (*N* = 419, twin 1 *n* = 203, twin 2 *n* = 216).

*df* = Degress of Freedom; χ^2^ = Chi square value for test of model fit estimated using Weighted Least Square Mean- and Variance-Adjusted estimator (WLSMV); *p* = significance value of the chi square test statistic; RMSEA = Root Mean Square Error of Approximation; *CI* = Confidence Interval; WRMR = Weighted Root Mean Square Residual; CFI = Comparative Fit Index; Δdf = delta degrees of freedom; Δχ^2^ = delta chi square; Δp = significance value of the delta chi square test statistic.

^1^Partial configural invariance – response category 4 was recoded as missing in twin 1 subsample for item 4 (*n* = 1), item 15 (*n* = 4), item 16 (*n* = 4 ), item 20 (*n* = 3), item 26 (*n* = 1), item 47 (*n* = 5); and item 41 (*n* = 1) in twin 2 subsample. ^2^Partial measurement invariance –the unstandardised loadings of item 11 on Lack of Premeditation (λ = 1.096, *p* < .001) and item 43 on Negative Urgency (λ = 1.189, *p* < .001) were freely estimated in twin 2 sample. ^3^Partial invariance of factor variances – the unstandardised variance of the Lack of Premeditation factor was freely estimated in the twin 2 sample to obtain model fit (σ^2^=.199, *p* = .001). ^4^Partial invariance of factor covariances – in the twin 2 subsample covariance between Sensation Seeking and Positive Urgency was freely estimated (θ_δ_ = .169, *p* = .004).

Table S7

*Summary of Fit Statistics for the Competing Confirmatory Factor Analysis Models for the Impulsivity and Compulsivity Phenotypes in the Twin 1 Subsample*

|  | Model | *df* | *χ*^2^ | *p* | RMSEA (90%*CI*) | CFI | SRMR |
| --- | --- | --- | --- | --- | --- | --- | --- |
| 1 | One-Factor ^1,2,3^ | 10 | 20.574 | .079 | .066 (.023 - .107) | .984 | .038 |
| 2 | Two-Factor ^1,2^ | 13 | 88.569 | <.001 | .155 (.125 - .186) | .902 | .126 |
| 3 | Three-Factor ^1,2,4^ | 15 | 103.501 | <.001 | .156 (.129 - .186) | .886 | .139 |
| 4 | Bi-Factor ^1^ | 17 | 30.359 | .083 | .057 (.021 - .089) | .983 | .064 |

*Note.* OBQ-44 = Obsessive Beliefs Questionnaire – 44-item version. *df* = Degress of Freedom; *χ*^2^ = Chi square value for test of model fit estimated using the Weighted Least Square Mean- and Variance-Adjusted estimator (WLSMV); *p =* significance value of the chi square test statistic; RMSEA = Root Mean Square Error of Approximation; *CI* = Confidence Interval; WRMR = Weighted Root Mean Square Residual; CFI = Comparative Fit Index; Δ*df* = delta degrees of freedom. Δ*χ*^2^ = delta chi square. Δ*p* = significance value of the delta chi square test statistic. *n* = 241.

^1^ Included freely estimated error covariances. ^2^ Sensation Seeking scale scores removed from model. ^3^ Lack of Perseverance scale scores removed the model. ^4^ Model estimated with Mplus version 8.3.

Table S8

*Intercorrelations Amongst the Subscale Scores Used as Indicator Variables in the Second-Order Overlapping Dimensional Phenotypes Model in the Twin Subsamples*

|  | 1 | 2 | 3 | 4 | 5 | 6 | 7 | 8 | 9 | 10 | 11 |
| --- | --- | --- | --- | --- | --- | --- | --- | --- | --- | --- | --- |
| 1. Age |  | .156  [.031, .276] | -.163  [-.286, -.035] | -.183  [-.304, -.056] | -.009  [-.138, .120] | -.093  [-.219, .036] | -.219  [-.344, -.086] | -.138  [-.269, -.002] | -.161  [-.281, -.036] | -.148 [-.269, -.023] | -.141 [-.262, -.015] |
| 2. Sex | .030  [-.097, .156] |  | .026  [-.104, .155] | .256  [.132, .372] | -.009  [-.138, .120] | .055 [-.074, .183] | -.052  [-.187, .085] | -.018  [-.154, .119] | .066  [-.060, .190] | .137  [.011, .258] | .109  [-.018, .232] |
| 3. Urgency | -.077  [-.207, .055] | .027  [-.105, .158] |  | .041  [-.089, .169] | .237  [.110, .356] | .376  [.259, .482] | .244  [.111, .368] | .381  [.258, .492] | .325  [0.204, .436] | .401  [.287, .504] | .430  [.318, .530] |
| 4. Sensation Seek | -.179  [-.304, -.048] | .279  [.152, .397] | -.007  [-.139, .126] |  | .283  [.160, .397] | -.088  [-.214, .041] | -.145  [-.275, .009] | -.223  [-.348, -.090] | .001  [-.128, .130] | -.022  [-.150, .107] | .039  [-.091, .167] |
| 5. Premeditation | .096  [-.036, .225] | -.036  [-.167, .096] | .273  [.146, .391] | .180  [.049, .305] |  | .393  [.278, .497] | -.416  [-.523, -.296] | -.290  [-.410, -.160] | -.268  [-.384, -.144] | -.174  [-.296, -.046] | -.085  [-.212, .045] |
| 6. Perseverance | -.025  [-.156, .106] | .119  [-.013, .247] | .468  [.358, .565] | -.120  [-.248, .012] | .418  [.303, .521] |  | -.122  [-.254, .014] | .105  [-.032, .238] | -.056  [-.183, .073] | .057  [-.072, .184] | .104  [-.026, .230] |
| 7. Predictability | -.211  [-.340, -.074] | -.107  [-.243, .033] | .160  [.021, .293] | -.132  [-.267, .008] | -.464  [-.567, -.346] | -.150  [-.284, -.011] |  | .635  [.546, .710] | .526  [.420, .618] | .509  [.401, .603] | .357  [.232, .471] |
| 8. Paralysis | -.232  [-.360, -.095] | -.006  [-.146, .135] | .232  [.094, .361] | -.182  [-.315, -.042] | -.267  [-.393, -.131] | .208  [.070, .339] | .571  [.468, .658] |  | .547  [.444, .636] | .561  [.460, .648] | .468  [.354, .569] |
| 9. Perfectionism | -.149  [-.271, -.023] | -.026  [-.153, .101] | .307  [.182, .422] | -.094  [-.224, .039] | -.344  [-.455, -.222] | -.121  [-.249, .011] | .554  [.449, .644] | .451  [.331, .557] |  | .737  [.674, .790] | .692  [.620, .752] |
| 10. Responsibility | -.156  [-.277, -.030] | .027  [-.100, .153] | .370  [.250, .479] | -.049  [-.180, .084] | -.231  [-.352, -.102] | -.021  [-.152, .111] | .480  [.365, .581] | .395  [.269, .507] | .735  [.671, .788] |  | .716  [.648, .772] |
| 11. Importance | -.122  [-.245, .005] | -.043  [-.170, .085] | .343  [.221, .455] | -.071  [-.202, .062] | -.092  [-.222, .041] | .090  [-.042, .219] | .412  [.289, .522] | .377  [.249, .492] | .622  [.537, .694] | .712 [.643, .769] |  |

*Note.* Twin 1 subsample (*n* = 241) = lower diagonal. Twin 2 subsample (*n* = 245) = upper diagonal. Age in years; Sex = Female (1); Male (2); Urgency = Summed Negative and Positive Urgency subscales (UPPS-P); Sensation Seek = Sensation Seeking subscale (UPPS-P); Premeditation = Lack of Premeditation subscale (UPPS-P); Perseverance = Lack of Perseverance subscale (UPPS-P); Predictability = Desire for Predictability and Control subscale (IUS-12); Paralysis = Paralysis of Cognition and Action in the Face of Uncertainty subscale (IUS-12); Perfectionism = Perfectionism subscale (OBQ-44); Responsibility = Responsibility and Threat Assessment subscale (OBQ-44); Importance = Importance and Control of Thoughts subscale (OBQ-44). 95%*CI* in brackets calculated for each subsample size ^24^.

Table S9

*Equivalence of Latent Means for the UPPS-P, IUS-12, and OBQ-44 Subscales in the Twin 1 and Twin 2 Subsamples*

| **Subscale** | **Δ*M*** | ***SE*** | ***Z*** | ***p*** |
| --- | --- | --- | --- | --- |
| UPPS-P |  |  |  |  |
| Lack of Premeditation | -.029 | .039 | -.733 | .463 |
| Negative Urgency | -.003 | .104 | -.025 | .980 |
| Sensation Seeking | .022 | .078 | .288 | .773 |
| Lack of Perseverance | -.031 | .095 | -.326 | .744 |
| Positive Urgency | .056 | .112 | .505 | .614 |
| IUS-12 |  |  |  |  |
| Desire for Predictability and an Active Engagement in Seeking Certainty | .134 | .126 | 1.062 | .288 |
| Paralysis of Cognition and Action in the Face of Uncertainty | -.143 | .219 | -.654 | .513 |
| OBQ-44 |  |  |  |  |
| Responsibility and Threat Estimation | -.011 | .057 | -.187 | .852 |
| Perfectionism | -.097 | .080 | -1.209 | .227 |
| Importance and Control of Thoughts | -.012 | .098 | -.120 | .905 |

*Note.* Δ*M* = Mean of Twin 1 subsample – Mean of Twin 2 subsample. SE = standard error of the difference. Z = Z statistic for comparison of means. *p* = two-tailed probability valued for the Z-test. UPPS-P = UPPS Impulsive Behavior Scale + Positive Urgency Measure (*N* = 471, twin 1 *n* = 230, twin 2 *n* = 241). OBQ-44 = Obsessive Beliefs Questionnaire – 44-item version (*N* = 495, twin 1 *n* = 241, twin 2 *n* = 254). IUS -12 = Intolerance of Uncertainty – 12-item version (*N* = 419, twin 1 *n* = 203, twin 2 *n* = 216).

Table S10

*Within-Trait and Cross-Trait Twin Correlations for the UPPS-P, IUS-12, and OBQ-44 Subscales in Monozygotic Twin Pairs*

|  | Age | Urge_2_ | Sens_2_ | Prem_2_ | Pers_2_ | Pred_2_ | Para_2_ | PC_2_ | RT_2_ | ICT_2_ |
| --- | --- | --- | --- | --- | --- | --- | --- | --- | --- | --- |
| 1.Age |  | -.128  [-.307, .060] | -.234  [-.403, -.050] | .019  [-.206, .169] | -.108  [-.289, .080] | -.240  [-.414, -.049] | -.156  [-.339, .039] | -.165  [-.336, .016] | -.232  [-.396, -.053] | -.144  [-.317, .039] |
| 2. Urge_1_ | -.033  [-.219, .155] | .269  [.081, .439] | -.114  [-.300, .080] | .017  [-.177, .210] | .198  [.006, .376] | .111  [-.092, .305] | .190  [-.011, .376] | .116  [-.073, .297] | .120  [-.069, .300] | .135  [-.054, .315] |
| 3. Sens_1_ | -.133  [-.312, .056] | -.130  [-.315, .064] | .566  [0.419, .684] | .088  [-.107, .277] | -.196  [-.374, .004] | -.114  [-.307, .089] | -.256  [-.434, -.059] | -.040  [-.226, .148] | .032  [-.156, .218] | .066  [-.124, .251] |
| 4. Prem_1_ | .091  [-.098, .274] | .066  [-.128, .255] | -.162  [-.344, .032] | .260  [.070, .432] | .060  [-.134, .250] | -.069  [-.266, .133] | -.099  [-.294, .104] | -.150  [-.328, .038] | -.100  [-.282, .089] | -.073  [-.258, .117] |
| 5. Pers_1_ | .070  [-.117, .252] | .175  [-.016, .354] | -.215  [-.390, .025] | .204  [.013, .381] | .455  [.278, .586] | -.067  [-.262, .133] | .102  [-.098, .294] | -.058  [-.241, .129] | -.006  [-.191, .180] | .034  [-.153, .219] |
| 6. Pred_1_ | -.267  [-.439, .076] | .023  [-.176, .220] | .116  [-.084, .307] | -.180  [-.366, .020] | -.122  [-.313, .078] | .356  [.162, .524] | .214  [.008, .402] | .204  [.010, .383] | .247  [.055, .421] | .178  [-.018, .361] |
| 7. Para_1_ | -.228  [-.407, -.032] | .148  [-.055, .339] | -.041  [-.241, .162] | -.140  [-.333, .064] | .109  [-.095, .304] | .272  [.068, .455] | .304  [.102, .482] | .220  [.024, .400] | .279  0.086, .452] | .178  [-.021, .364] |
| 8. PC_1_ | -.185  [-.355, -.003] | .052  [-.137, .237] | .038  [-.151, .225] | -.223  [-.395, -.036] | -.086  [-.270, .104] | .314  [.127, .479] | .266  [.075, .438] | .357  [.187, .506] | .250  [.071, .413] | .231  [.050, .397] |
| 9. RT_1_ | -.187  [-.356, -.006] | .217  [.032, .388] | -.004  [-.191, .183] | -.055  [-.241, .134] | .011  [-.177, .198] | .308  [.122, .473] | .347  [.165, .507] | .347  [.177, .497] | .385  [.219, .530] | .286  [.109, .445] |
| 10. ICT_1_ | -.096  [-.274, .089] | .190  [.002, .365] | .009  [-.180, .198] | -.002  [-.192, .188] | .005  [-.184, .194] | .237  [.044, .413] | .269  [.078, .441] | .345  [.173, .497] | .239  [.058, .404] | .349  [.176, .501] |

*Note.* MZ pairs (*n* = 115) = lower diagonal. Age in years; Urge = Summed Negative and Positive Urgency subscales (UPPS-P); Sen = Sensation Seeking subscale (UPPS-P); Prem = Lack of Premeditation subscale (UPPS-P); Pers = Lack of Perseverance subscale (UPPS-P); Pred = Desire for Predictability and Control subscale (IUS-12); Para = Paralysis of Cognition and Action in the Face of Uncertainty subscale (IUS-12); PC = Perfectionism subscale (OBQ-44); RT = Responsibility and Threat Estimation subscale (OBQ-44); ICT = Importance and Control of Thoughts subscale (OBQ-44). 95%*CI* in brackets calculated for each subsample size ^24^.

Table S11

*Within-Trait and Cross-Trait Twin Correlations for the UPPS-P, IUS-12, and OBQ-44 Subscales in Dizygotic Twin Pairs*

|  | Age | Urge_2_ | Sens_2_ | Prem_2_ | Pers_2_ | Pred_2_ | Para_2_ | PC_2_ | RT_2_ | ICT_2_ |
| --- | --- | --- | --- | --- | --- | --- | --- | --- | --- | --- |
| 1.Age |  | -.155  [-.411, .123] | -.048  [-.312, .223] | -.009  [-0.276, .259] | -.154  [-.405, .119] | -.180  [-.436, .104] | -.115  [-0.384, .172] | -.141  [-0.394, .132] | -.110  [-.367, .163] | .010  [-.261, .280] |
| 2. Urge_1_ | -.188  [-.434, .084] | .128  [-.153, .390] | -.187  [-.435, .088] | -.174  [-.424, .101] | .156  [-.119, .409] | -.057  [-.330, .225] | .131  [-.156, .398] | .122  [-.153, .380] | .159  [-.116, .412] | .087  [-.190, .352] |
| 3. Sens_1_ | -.148  [-.400, .125] | -.007  [-.282, .269] | .301  [.033, .528] | .120  [-.155, .378] | -.030  [-.298, .242] | -.068  [-.340, .214] | -.135  [-.401, .152] | .128  [-0.147, .385] | .030  [-.242, .298] | -.025  [-.296, .250] |
| 4. Prem_1_ | -.029  [-0.294, .241] | -.193  [-.445, .087] | .010  [-.261, .280] | -.061  [-.326, .213] | -.005  [-.275, .266] | -.196  [-.450, .087] | -.123  [-.391, .164] | -.066  [-.330, .208] | -.198  [-.445, .076] | -.150  [-.406, .128] |
| 5. Pers_1_ | -.180  [-.427, .092] | -.077  [-.345, .203] | -.173  [-.423, .102] | -.169  [-.420, .106] | .257  [-.014, .493] | -.267  [-.508, .012] | .033  [-.251, .311] | -.122  [-.380, .153] | -.069  [-.333, .205] | .019  [-.255, .290] |
| 6. Pred_1_ | -.059  [-.327, .217] | -.054  [-.330, .231] | -.024  [-.298, .253] | -.032  [-.305, .246] | -.173  [-.428, .108] | .304  [.018, .544] | .038  [-.255, .325] | .095  [-.185, .361] | .171  [-.110, .426] | .178  [-.106, .435] |
| 7. Para_1_ | -.231  [-.474, .045] | .084  [-.202, .357] | -.287  [-.521, .012] | -.047  [-.318, .232] | .085  [-.195, .352] | .222  [-.070, .479] | .290  [.000, .535] | .267  [-.009, .505] | .332  [.062, .557] | .318  [.043, .548] |
| 8. PC_1_ | -.050  [-.311, .218] | .188  [-.089, .438] | -.083  [-.343, .189] | .003  [-.265, .271] | -.037  [-.302, .233] | .413  [.152, .620] | .321  [.044, .552] | .303  [.038, .528] | .315  [.052, .537] | .255  [-.016, .491] |
| 9. RT_1_ | -.123  [-.376, .147] | .095  [-.183, .359] | -.051  [-.314, .220] | -.013  [-.265, .271] | .058  [-.213, .321] | .308  [.032, .540] | .190  [-.096, .447] | .320  [.057, .541] | .375  [.119, .584] | .182  [-.093,.431] |
| 10. ICT_1_ | -.109  [-.364, .161] | .207  [-.07, .454] | -.043  [-.307, .227] | -.011  [-.278, .258] | .179  [-.093, .426] | .374  [.107, .591] | .266  [-.016, .509] | .237  [-.033, .475] | .295  [.030, .522] | .221  [-.052, .464] |

*Note.* DZ pairs (*n* = 55). Age in years; Urge = Summed Negative and Positive Urgency subscales (UPPS-P); Sen = Sensation Seeking subscale (UPPS-P); Prem = Lack of Premeditation subscale (UPPS-P); Pers = Lack of Perseverance subscale (UPPS-P); Pred = Desire for Predictability and Control subscale (IUS-12); Para = Paralysis of Cognition and Action in the Face of Uncertainty subscale (IUS-12); PC = Perfectionism subscale (OBQ-44); RT = Responsibility and Threat Estimation subscale (OBQ-44); ICT = Importance and Control of Thoughts subscale (OBQ-44). 95%*CI* in brackets calculated for each subsample size ^24^.

**A) B)**

**
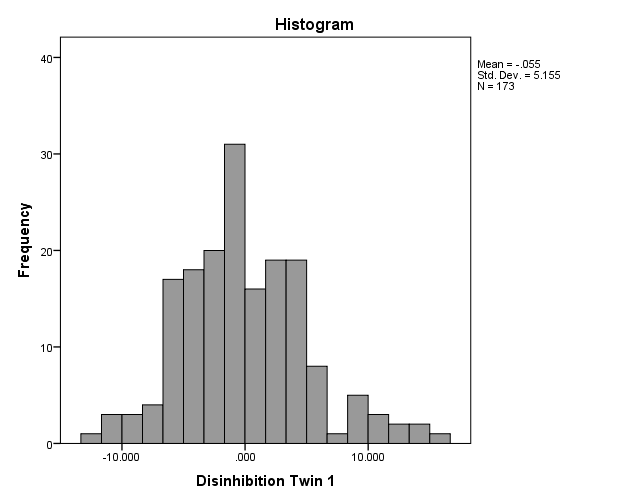

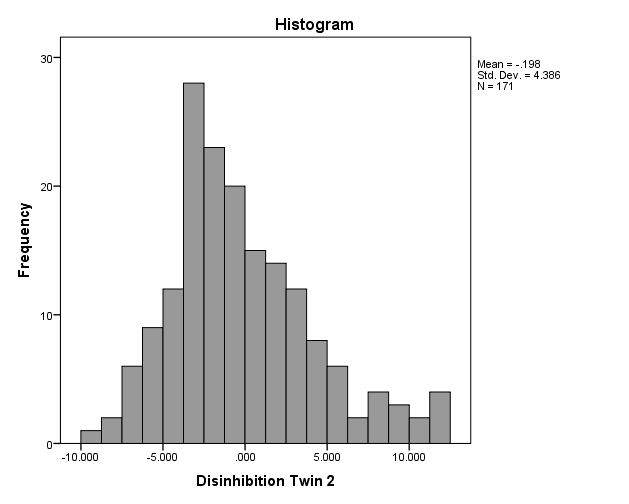
**

**Impulsive-Compulsive Factor Score Estimates Twin 1 Impulsive-Compulsive Factor Score Estimates Twin 2**

*Figure S11.* Distribution of Factor Score Estimates for the General Impulsive-Compulsive Phenotype in the A) Twin 1 and B) Twin 2 Subsamples.

*Note.* Factor score estimates are standardised with reference to the sample and therefore have a mean of zero and a standard deviation of one. Twin 1 *n* = 173 (*M* = -.055, *SD* = 5.16). Twin 2 *n* = 171 (*M* = -.198, *SD* = 4.386)

**A) B)**

**
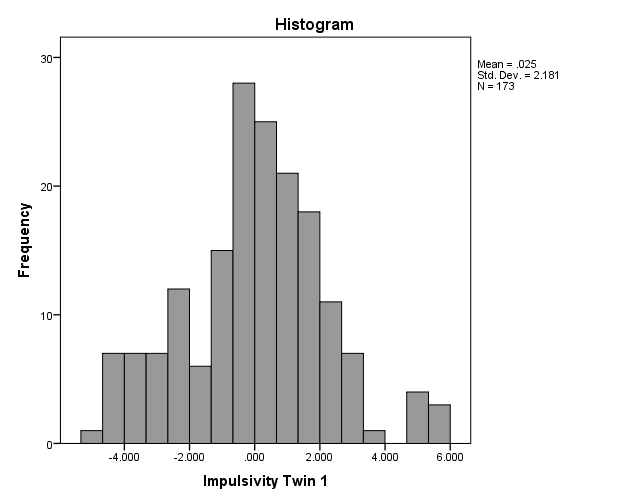

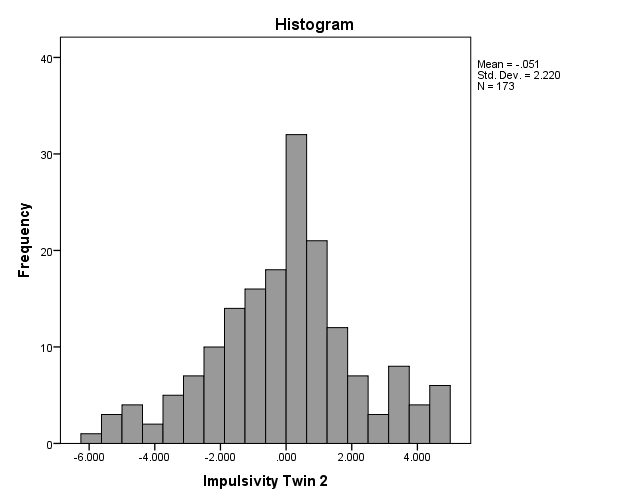
**

**Impulsivity Factor Score Estimates Twin 1 Impulsivity Factor Score Estimates Twin 2**

*Figure S12.* Distribution of Factor Score Estimates for the Impulsivity Phenotype in the A) Twin 1 and B) Twin 2 Subsamples.

*Note.* Factor score estimates are standardised with reference to the sample and therefore have a mean of zero and a standard deviation of one. Twin 1 *n* = 173 (*M* = .025, *SD* = 2.18). Twin 2 *n* = 173 (*M* = -.051, *SD* = 2.22)

**A) B)**

**
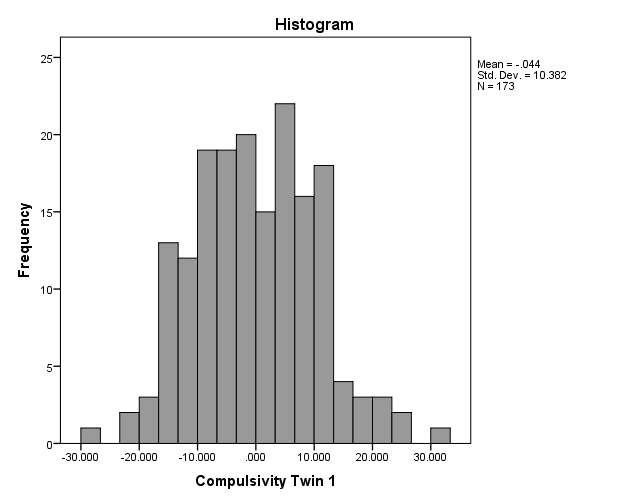

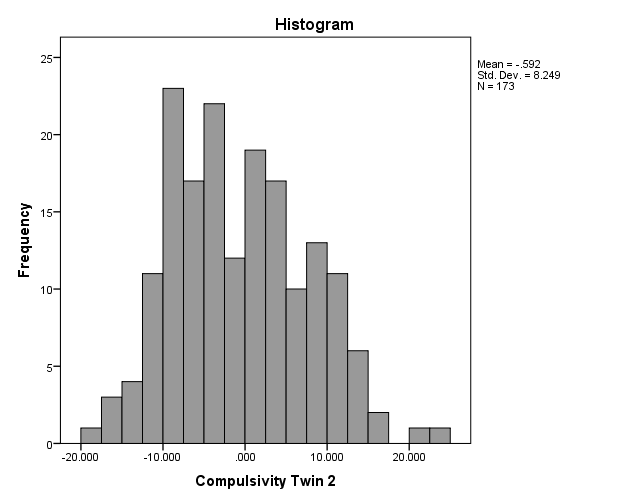
**

**Obsessiveness Factor Score Estimates Twin 1 Obsessiveness Factor Score Estimates Twin 2**

*Figure S13*. Distribution of Factor Score Estimates for the Obsessiveness Phenotype in the A) Twin 1 and B) Twin 2 Subsamples.

*Note.* Factor score estimates are standardised with reference to the sample and therefore have a mean of zero and a standard deviation of one. Twin 1 *n* = 173 (*M* = -.044, *SD* = 10.38). Twin 2 *n* = 173 (*M* = -.051, *SD* = 2.22).

**REFERENCES**

1 Guo, K. *et al.* A psychometric validation study of the Impulsive-Compulsive Behaviours Checklist: A transdiagnostic tool for addictive and compulsive behaviours. *Addict Behav* **67**, 26-33, doi:10.1016/j.addbeh.2016.11.021 (2017).

2 Patton, J. H., Stanford, M. S. & Barratt, E. S. Factor structure of the Barratt impulsiveness scale. *Journal of clinical psychology* **51**, 768-774, doi:<https://doi.org/10.1111/j.2044-8260.1982.tb01421.x> (1995).

3 Foa, E. B. *et al.* The Obsessive-Compulsive Inventory: Development and validation of a short version. *Psychological Assessment* **14**, 485 - 496, doi:10.1037//1040-3590.14.4.485 (2002).

4 Holtgraves, T. Evaluating the problem gambling severity index. *Journal of Gambling Studies* **25**, 105 - 120, doi:<https://doi.org/10.1007/s10899-008-9107-7> (2009).

5 Ferris, J. A. & Wynne, H. J. *The Canadian problem gambling index*. 1-59 (Canadian Centre on Substance Abuse 2001).

6 Carver, C. S. & White, T. L. Behavioral inhibition, behavioral activation, and affective responses to impending reward and punishment: the BIS/BAS scales. *Journal of personality and social psychology* **67**, 319, doi:<http://dx.doi.org/10.1037/0022-3514.67.2.319> (1994).

7 Bernstein, D. P. *et al.* Initial reliability and validity of a new retrospective measure of child abuse and neglect. *Am J Psychiatry* **151**, 1132-1136, doi:10.1176/ajp.151.8.1132 (1994).

8 Endicott, J., Nee, J., Harrison, W. & Blumenthal, R. Quality of life enjoyment and satisfaction questionnaire: A new measure. *Psychopharmacology Bulletin* **29**, 321-326 (1993).

9 Stevanovic, D. Quality of Life Enjoyment and Satisfaction Questionnaire – short form for quality of life assessments in clinical practice: a psychometric study. *Journal of Psychiatric and Mental Health Nursing* **18**, 744-750, doi:10.1111/j.1365-2850.2011.01735.x (2011).

10 Little, R. J. A. A test of missing completely at random for multivariate data with missing values. *Journal of the American Statistical Association* **83**, 1198 - 1202 (1988).

11 Enders, C. K. *Applied missing data analysis.*, (The Guilford Press, 2010).

12 Hoaglin, D. C. & Iglewicz, B. Fine-tuning some resistant rules for outlier labeling. *Journal of the American Statistical Association* **82**, 1147 - 1149 (1987).

13 Tabachnick, B. G. & Fidell, L. S. *Using multivariate statistics.* 6th edn, (Pearson Education, Inc., 2013).

14 DeCarlo, L. T. On the meaning and use of kurtosis. *Psychological Methods* **2**, 292 - 307 (1997).

15 Small, N. Marginal skewness and kurtosis in testing multivariate normality. *Applied Statistics*, 85-87 (1980).

16 Tiego, J. *et al.* Overlapping dimensional phenotypes of impulsivity and compulsivity explain co-occurrence of addictive and related behaviors. *CNS Spectrums*, 1-15, doi:10.1017/S1092852918001244 (2018).

17 Streiner, D. L. Starting at the beginning: An introduction to coefficient alpha and internal consistency. *Journal of Personality Assessment* **80**, 99 103, doi:10.1207/s15327752jpa8001_18 (2003).

18 Toland, M. D. Practical guide to conducting an item response theory analysis. *The Journal of Early Adolescence* **34**, 120-151, doi:10.1177/0272431613511332 (2014).

19 Vandenberg, R. J. Toward a further understanding of and improvement in measurement invariance methods and procedures. *Organizational Research Methods* **5**, 139-158, doi:10.1177/1094428102005002001 (2002).

20 Byrne, B. M., Shavelson, R. J. & Muthén, B. Testing for the equivalence of factor covariance and mean structures: The issue of partial measurement invariance. . *Psychol Bull* **105**, 456 - 466, doi:<http://dx.doi.org/10.1037/0033-2909.105.3.456> (1989).

21 Kline, R. B. *Principles and practice of structural equation modeling*. 4th edn, (The Guilford Press, 2015).

22 OCCWG. Psychometric validation of the Obsessive Beliefs Questionnaire and the Interpretation of Intrusions Inventory. Part 2: Factor analyses and testing of a brief version. *Behaviour Research and Therapy* **43**, 1527 –1542, doi:<https://doi.org/10.1016/j.brat.2004.07.010> (2005).

23 Myers, S. G., Fisher, P. L. & Wells, A. Belief domains of the Obsessive Beliefs Questionnaire-44 (OBQ-44) and their specific relationship with obsessive-compulsive symptoms. *J Anxiety Disord* **22**, 475 - 484, doi:<https://doi.org/10.1016/j.janxdis.2007.03.012> (2008).

24 Lenhard, W. & Lenhard, A. *Hypothesis tests for comparing correlations [*[*https://www.psychometrica.de/correlation.html*](https://www.psychometrica.de/correlation.html)*]*. (Psychometrica, 2014).
